# CORE: Cholesterol Altered Lipid Nanoparticles for Splenic Expression of mRNA Payloads

**DOI:** 10.1002/adhm.202505862

**Published:** 2026-03-28

**Authors:** Eshan A. Narasipura, Vincent Fung, Rachel VanKeulen‐Miller, Palas B. Tiwade, Owen S. Fenton

**Affiliations:** ^1^ Division of Pharmacoengineering and Molecular Pharmaceutics Eshelman School of Pharmacy University of North Carolina At Chapel Hill Chapel Hill North Carolina USA; ^2^ Department of Pharmacology School of Medicine University of North Carolina at Chapel Hill Chapel Hill North Carolina USA

**Keywords:** extrahepatic drug delivery, material synthesis, mRNA

## Abstract

A central challenge within the field of nanomedicine is synthesizing and formulating vectors capable of extrahepatic delivery. mRNA lipid nanoparticles (LNPs) are no exception as most lipid‐based formulations, when administered systemically, facilitate delivery and expression of mRNA to the liver. To address this delivery challenge, we developed a new class of LNPs—termed **C**h**O**lesterol alte**RE**d lipid nanoparticles (**CORE** LNPs)—designed to shift mRNA expression away from the liver and toward the spleen, a central organ for systemic immune activation. To do so, we interfaced material synthesis with computational characterization to develop a series of novel cholesterol analogs which were incorporated alongside formulation components used for mRNA delivery. These were then optimized through design of experiment to generate 78 discrete formulations and were assessed for their physical properties, cellular uptake, and endosomal escape capabilities in vitro. Importantly, in vivo studies demonstrated that **CORE** LNPs were well‐tolerated and achieved highly selective mRNA expression in the spleen following intravenous administration. These results suggest that interfacing synthesis with computational characterization of novel cholesterol derivatives may modulate both nanoparticle behavior and organ‐level targeting, offering a promising strategy for developing spleen‐specific mRNA therapies for vaccination and immune modulation.

## Graphical abstract

In this paper researchers introduce **CORE** LNPs, a new class of lipid nanoparticles engineered to redirect mRNA expression away from the liver and into the spleen, a key immune organ. By combining chemical design with computational tools, they created cholesterol analogs that enable precise spleen‐targeted expression, providing greater applications for mRNA‐based therapies.

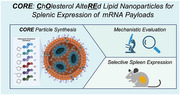

## 1 Introduction

mRNA‐based therapies have advanced as a potential strategy to treat various diseases due to their ability to transiently express proteins in a controlled, cell‐specific manner [1, 2, 3, 4]. While this modularity invites an exciting potential for treating a range of conditions—from cancer to genetic and autoimmune diseases—the physicochemical properties of mRNA prevent effective delivery when administered in vivo [5, 6, 7, 8, 9]. Specifically, its tendency to be degraded by RNases, large size, and negative charge prevent efficient delivery and cellular uptake [10, 11, 12, 13, 14]. These challenges associated with mRNA have necessitated the development of several delivery strategies for effective mRNA translation for the overall therapeutic success of mRNA‐based therapies [15, 16, 17, 18].

One such delivery strategy which has shown success in the delivery of mRNA in vivo are LNPs which have enabled for clinical translation of mRNA therapies [5, 19, 20, 21, 22]. However, a central challenge of LNPs is extrahepatic delivery after intravenous injection [23, 24, 25, 26]. Development of mRNA carriers which can deliver and express mRNA in organs excluding the liver may provide more therapeutic opportunities for mRNA‐based therapies [27, 28]. One such organ which is relevant in the context of several diseases is the spleen. Work has shown that expression of mRNA encoding antigens to the spleen can enhance antigen‐specific T cell activation and generate stronger type I interferon responses compared to other lymphoid sites [29, 30, 31, 32, 33]. This is potentially due to the spleen's central role in systemic immune coordination, as it hosts a dense population of antigen‐presenting cells and direct access to blood‐borne pathogens [34, 35]. These immunological features have made the spleen an attractive target for mRNA‐based vaccines and immune therapies [36, 37, 38]. Motivated by this evidence, we aimed to design an mRNA platform that could efficiently target the spleen following systemic administration.

To develop this platform, we specifically investigated cholesterol and its role in dictating liver tropism. It has been shown that the lipoprotein ApoE is involved in cholesterol transport to the liver within the bloodstream [39, 40, 41, 42, 43, 44]. Thus, we hypothesized that by structurally modifying cholesterol we could affect this cholesterol‐dependent transport, potentially shifting mRNA delivery and expression toward the spleen. Not only did we seek to modify cholesterol as a strategy to modulate biodistribution, but we also sought to better understand how adding structural motifs to cholesterol, specifically nitrogen‐based heterocyclic motifs, can impact key mechanistic steps important to mRNA expression. Nitrogen‐based heterocyclic motifs have gained attention due to their immune modulating properties [45, 46]. We sought to synergize these structural findings with cholesterol's unique role in LNP function to synthesize and characterize novel materials for mRNA delivery.

**FIGURE 1:**
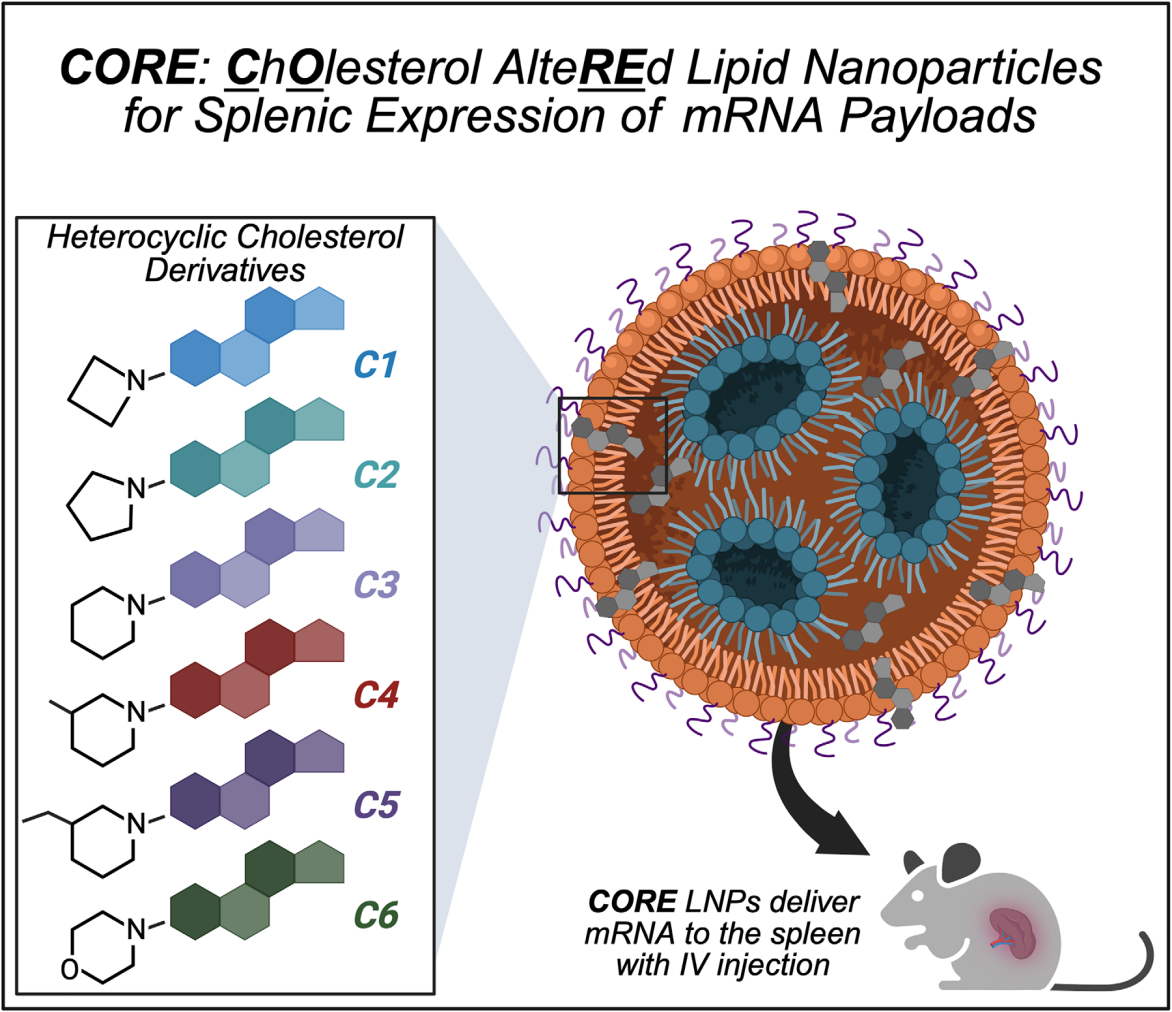
Development of heterocyclic cholesterol derivatives enabling the development of **CORE** LNPs for mRNA expression in the spleen.

Upon the synthesis and computational characterization of six heterocyclic cholesterol derivatives, we formulated each of these novel materials alongside lipids commonly used for mRNA LNPs (ionizable lipids, phospholipids, and PEG‐lipids) to form **C**h**O**lesterol alte**RE**d (**CORE**) particles (Figure 1). These base particle components were then further optimized via design of experiment to identify six lead **CORE** LNP formulations. These optimized **CORE** LNPs were then further studied for their in vitro cellular uptake properties, cellular uptake mechanism, and endosomal escape properties. For these in vitro mechanistic studies, we utilized a dendritic cell model (DC 2.4 cells) as dendritic cells play an important role in mRNA‐based immunotherapies—specifically via antigen presentation and co‐stimulation for the generation of cytotoxic CD8+ T cells [47, 48, 49, 50, 51, 52, 53]. From these mechanistic studies, we were able to understand how our novel heterocyclic cholesterol derivatives can impact both particle characteristics and mRNA performance. Finally, we validated our findings in vivo, demonstrating that our lead **CORE** LNPs were well tolerated—based on full blood paneling and histological analysis of the liver, lung, and spleen—and exhibited nearly 100% splenic expression upon IV administration, as indicated by IVIS imaging of harvested organs. Taken together, our approach highlights the utility of synthesizing and characterizing novel materials for the development of mRNA carriers capable of mRNA delivery to important immunological organs like the spleen.

## 2 Results

To begin developing a series of spleen expressing lipid nanoparticles, we sought to utilize a synthetic chemistry‐based approach paired with computational characterization and in vitro mechanistic studies to develop a platform capable of mRNA delivery and expression to the spleen. To begin, we identified cholesterol out of the four components of the classical LNP framework to structurally modify to shift organ tropism. Cholesterol's structure, specifically the sterol rings and tail structure, has been shown to readily change LNP properties from stiffness, lamellarity, endosomal escape, and in vivo tropism [23, 54, 55, 56, 57, 58]. Additionally, naturally occurring cholesterol derivatives such as β‐sitosterol have been utilized in mRNA LNPs [59, 60]. However, the addition of structural motifs, specifically the incorporation of an additional ring group, via cholesterol's alcohol head group have not been readily studied. Thus, we started by adding a series of heterocyclic ring groups via cholesterol's alcohol functional group given heterocyclic rings can play a role in immune modulation while also having the ability to alter physical properties of cholesterol thus promoting mRNA expression to the spleen [61, 62, 63]. To further complement our synthesis, each cholesterol derivative was computationally characterized for 23 physicochemical parameters to better understand how these derivatives may behave in 3D space given each derivative interacts within other components upon formulation with mRNA.

**FIGURE 2:**
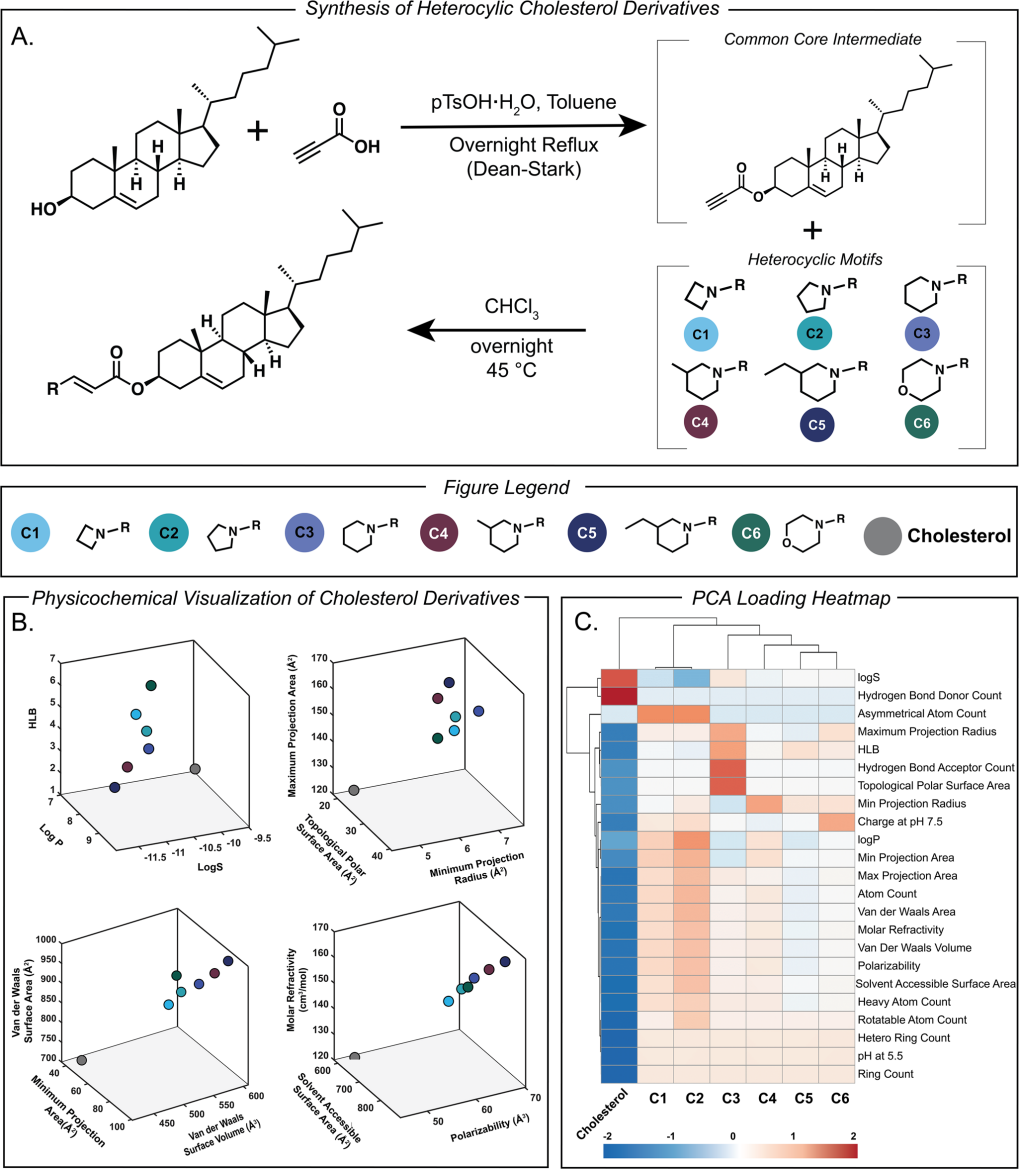
(a) Reaction scheme of the synthesis of six heterocyclic cholesterol derivatives termed **C1‐6** via synthesis of common core intermediate. (b) Computational characterization of **C1‐6** and cholesterol measuring respective physicochemical properties visualized in three dimensions. (c) PCA loading heatmap of **C1‐6** and cholesterol.

We began the synthesis of our heterocyclic cholesterol derivatives through the use of a common core approach where cholesterol was reacted with propiolic acid to form a common core intermediate ester based cholesterol derivative (Figure 2A; Figure S1). From this common core we utilized six nitrogen containing heterocycles (Azetidine, Pyrrolidine, Piperidine, 3‐Methylpiperidine, 3‐Ethylpiperidine, and Morpholine) which were reacted to form six heterocyclic cholesterol derivatives termed **C1‐6** (Figure 2A; Figures S2–S7). Upon synthesis, **C1‐6** and cholesterol were measured for 23 physicochemical properties which were measured via ChemAxon and subsequently plotted onto 2D space utilizing a principal component analysis (PCA) plot followed by a heatmap loading showing how each of the 23 physicochemical parameters contributes (positively or negatively) to each principal component (strong positive or negative loadings mean that parameter has a larger influence on positioning samples along the respective PC axis). Upon computational characterization, several findings emerged: first, it was found **C1‐6** were structurally similar as a function of logS, minimum/maximum projection radius and surface area, polarizability, and molar refractivity (Figure 2B; Figure S8). Second, when all 23 physicochemical properties for **C1‐6** and cholesterol were plotted into two dimensions utilizing a PCA plot, it was found **C1‐6** clustered more closely together as compared to cholesterol indicating **C1‐6** were more structurally similar to each other as compared to cholesterol itself (Figure S9). To further understand what properties contributed to the separation seen in the PCA plot, we utilized a heatmap to show how each physicochemical parameter measured contributed to the relative positive and negative loading of the PCA axis. It was found cholesterol showed positive loadings with hydrophobic traits (logP, van der Waals volume, molar refractivity, and polarizability) and negative loadings with polar traits (hydrogen bond counts and topological polar surface area). This loading indicates that cholesterol primarily was separated within the PCA plot along a hydrophobic–polar axis, favoring lipophilic molecular properties as compared to **C1‐6** (Figure 2C).

**FIGURE 3:**
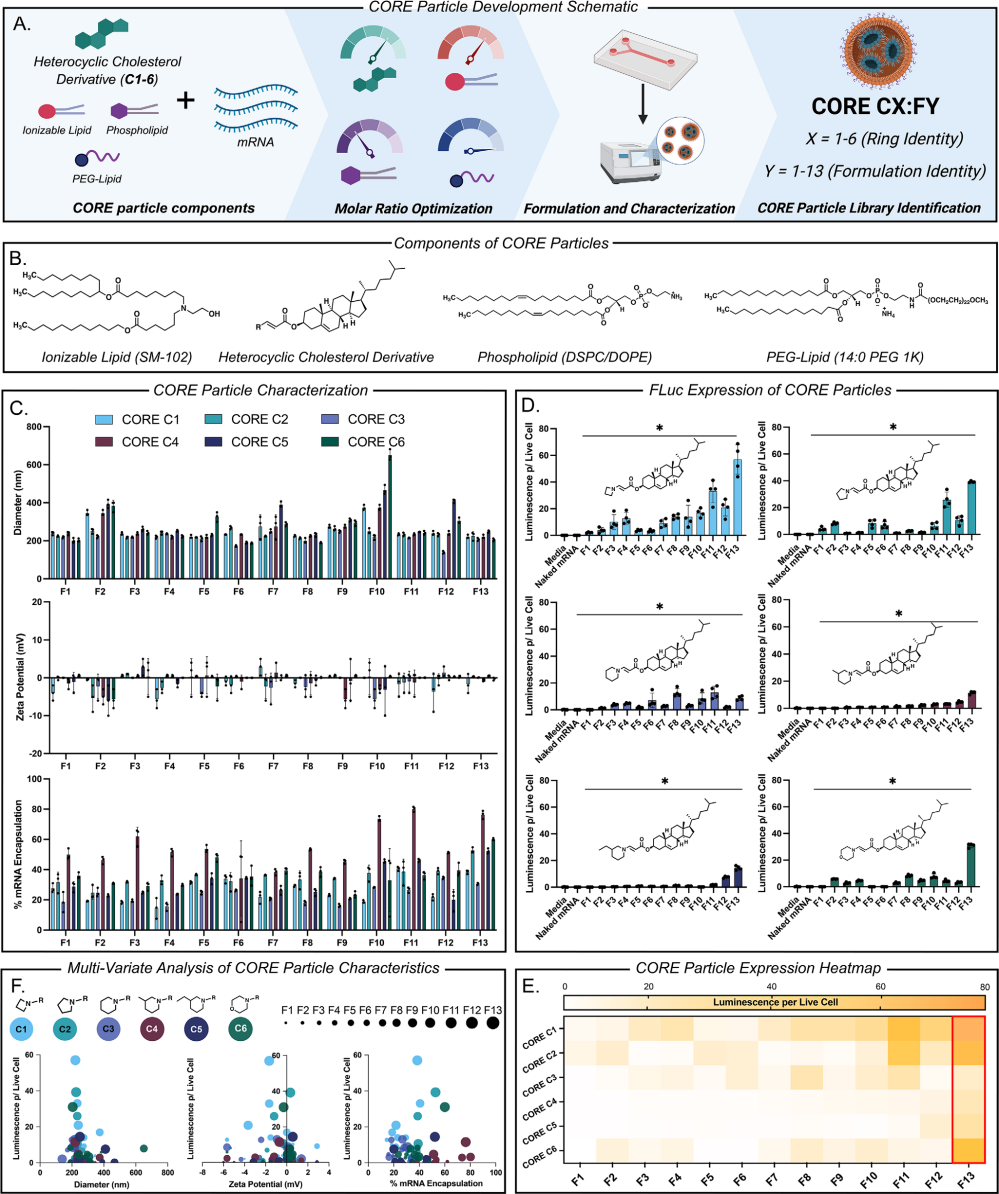
(a) Schematic workflow for **CORE** LNP formulation development. (b) Chemical structures of the components used to form **CORE** LNPs where each particle contains SM‐102, its respective cholesterol derivative (**C1‐6**), DSPC or DOPE, and 14:0 PEG 1K. (c) **CORE** LNP size (nm), charge (mV), and % mRNA encapsulation for each respective formulation. (d) 24 h FLuc expression of respective **CORE** LNPs treated on DC 2.4 cells at a dose of 50 ng FLuc mRNA. (e) Heat map representation of FLuc expression for all 78 **CORE** LNP formulations. (f) Multivariate analysis of **CORE** LNP expression as a function of size, charge, and % mRNA encapsulation. (All data represented as the mean ± standard deviation, *n*=4, ^*^ *p*<0.05 as compared to naked mRNA treatment group using one‐way ANOVA, Dunnett's test).

Having characterized the structures of **C1‐6** we began formulating particles with the overall goal of maximizing mRNA delivery and expression in cells. To do so, we utilized a systematic development process where lipid components were formulated alongside compounds **C1‐6** via microfluidics to form particles, broadly termed **C**h**O**lesterol alte**RE**d (**CORE**) particles (Figure 3A). Specific nomenclature of each **CORE** LNP is denoted via the identity of its respective cholesterol derivative followed by the formulation ratio—i.e. **CORE C1:F1** indicates the incorporation of cholesterol derivative **C1** into the **F1** formulation ratio (Figure 3A). To begin this process, we decided to utilize well characterized LNP components alongside our novel cholesterol derivatives for successful particle formation and efficient mRNA delivery. We chose to use the ionizable lipid SM‐102, phospholipids DSPC or DOPE, and the PEG‐lipid 14:0 PEG 1K as these materials have been previously shown to be effective in both particle formation and mRNA complexation and delivery (Figure 3B) [64, 65, 66]. Given that we had developed new materials, it was then necessary to identify an optimal formulation ratio of all four lipid components for effective mRNA delivery and expression. To identify this optimized formulation, we employed design of experiment to pinpoint an optimal lipid ratio by testing multiple formulation ratios within a large formulation design space (Figure S10) [67]. Specifically, we utilized a definitive screening design with the molar parameters of 35%–70% ionizable lipid, 20%–55% respective cholesterol derivative, 3%–21% phospholipid, and 0.4%–2.5% PEG‐lipid. Upon using design of experiment, we generated a total of 13 base formulation ratios denoted **FX** (where **X** represents the formulation identity) (Figure S11). Having generated 13 formulation ratios, we then individually formulated the six cholesterol derivatives alongside the three other components to generate a total of 78 discrete **CORE** LNPs. All these formulations were synthesized with Firefly luciferase mRNA and characterized for their size, charge, mRNA encapsulation percentage, and FLuc protein expression on DC 2.4 cell lines. We utilized an in vitro dendritic cell model as one potential target cell type for mRNA vaccines given presentation may be a part of immune cascades. Although T cells and B cells may also be present in the spleen, we aimed to target dendritic cells to further elucidate particle properties and delivery. Upon analyzing these formulations, several findings emerged. To begin, we found all 78 formulations successfully formed particles ranging from 190 – 400 nm in size, −10 ‐ +5 mV in charge, and were all effective in encapsulating mRNA with a range of 20%–85% mRNA encapsulation (Figure 3C). Second, **CORE** LNPs utilizing **C1** had the lowest average diameters across the thirteen formulations, while **CORE** LNPs utilizing **C4** had the highest average mRNA encapsulation across all thirteen formulations (Figure 3C). Third, **CORE C1** had the highest average FLuc expression across all formulations followed by **C2** and **C6,** which may be attributed to the differences in the head group motifs of each cholesterol derivative associated with each **CORE** LNP coupled with **CORE** LNP characteristics specifically particle size and encapsulation efficiency (Figure 3D). Furthermore, **CORE C3, 4,** and **5** had lower expression across all thirteen formulations as compared to **1, 2,** and **6,** suggesting an increase in the ring size of each cholesterol derivative may contribute to the decrease in **CORE** LNP performance (Figure 3D). Additionally, **F13** was the highest performing formulation across all six **CORE** LNPs, all exhibiting significantly higher luminescence as compared to naked mRNA, leading us to choose **F13** as a lead formulation in our future studies (Figure 3D,E). Upon multivariate analysis of **CORE** LNP characteristics, it was also found that expression was more closely correlated with the identity of the cholesterol derivative and formulation ratio than the size, charge, and mRNA encapsulation of each LNP (Figure 3F). Lastly, all formulations were well tolerated as compared to a negative “kill control” averaging 80%–100% viability 24 h after treatment on DC 2.4 cells (Figure S12). We also assessed **CORE** LNP morphology via transmission electron microscopy (TEM) (Figure S13). To further investigate the performance of the lead **CORE** formulation **F13** on other relevant immune cells residing within the spleen, we dosed all six lead particles on T cells, specifically Jurkat cells. It was found the performance (as quantified by FLuc expression) of each formulation was statistically significant as compared to delivery of naked mRNA (Figure S14). These results suggest that **CORE** LNPs may be used to deliver mRNA into T cells both ex vivo and in situ to the spleen in addition to cancer vaccines which require mRNA delivery to antigen presenting cells.

**FIGURE 4:**
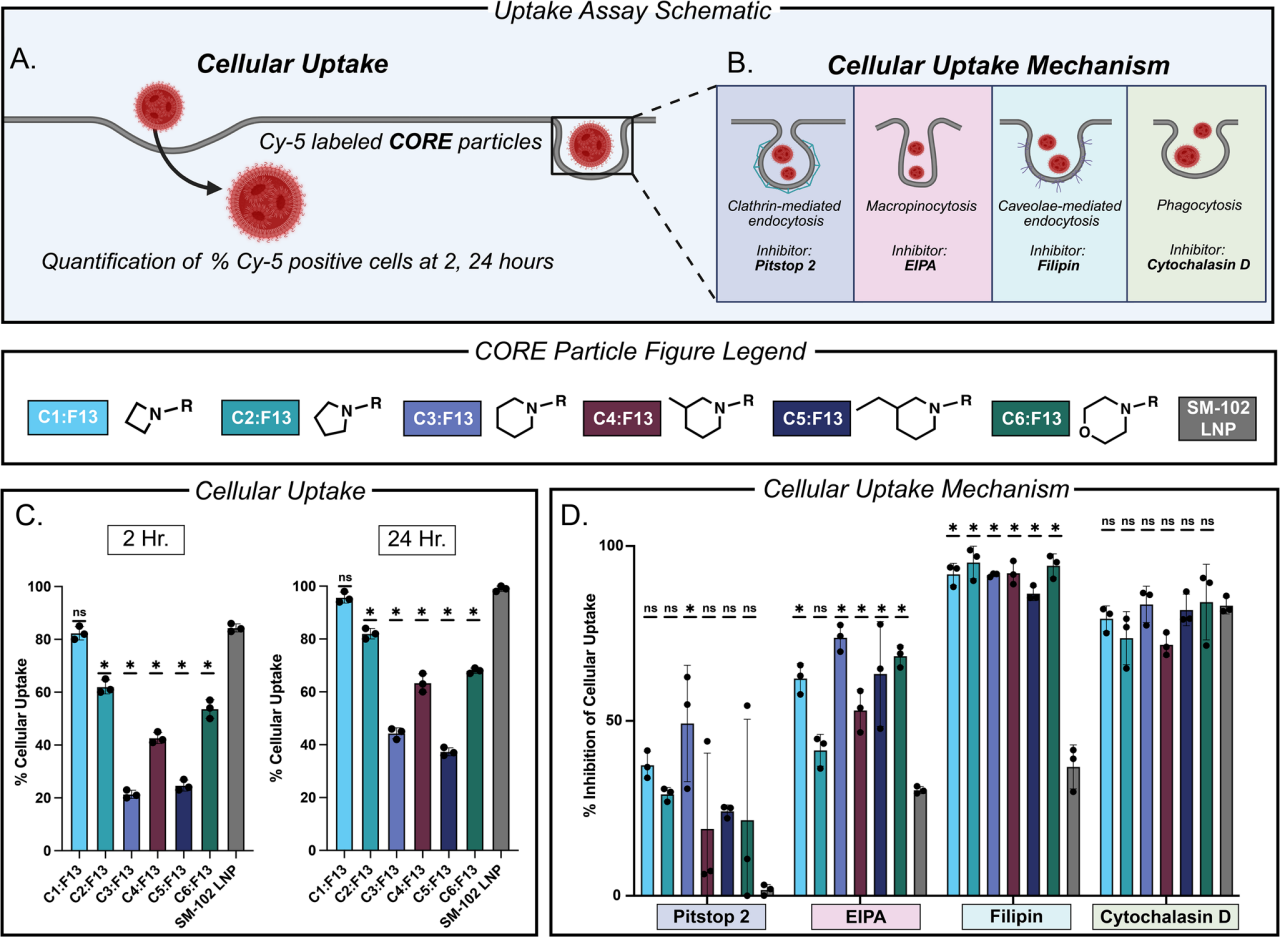
(a) Schematic overview of cellular uptake assay. (b) Schematic overview of cellular uptake mechanism assay. (c) Evaluation of cellular uptake of **CORE** LNPs encapsulating Cy‐5 labeled FLuc mRNA at 2 and 24 h on DC 2.4 cells. (d) Elucidation of the cellular uptake mechanism of respective **CORE** LNPs using inhibitors: Pitstop 2 (clathrin‐mediated endocytosis), EIPA (macropinocytosis), filipin (caveolae‐mediated endocytosis), and cytochalasin D (phagocytosis) treated on DC 2.4 cells. (All data represented as the mean ± standard deviation, *n* = 4). ^*^ *p*<0.05 as compared to SM‐102 LNP treatment group using one‐way ANOVA, Dunnett's test).

Following screening and lead formulation for the six cholesterol derivatives, we began several mechanistic studies for each lead **CORE** LNP. Specifically, we evaluated cellular uptake which is important for determining which lead **CORE** LNP identity is readily taken up by dendritic cells over time, cellular uptake mechanism which is important for understanding how exactly dendritic cells recognize lead **CORE** LNP, and endosomal escape which is important for understanding a key rate limiting step in mRNA efficacy. To begin, we evaluated the cellular uptake of each **CORE** LNP taken up by DC 2.4 cells at 2 and 24 h followed by the uptake mechanism by which these **CORE** LNPs are taken up (Figure 4A). Uptake mechanisms were investigated by inhibiting specific endocytosis pathways individually using pathway‐specific inhibitors: Pitstop 2 for clathrin‐mediated endocytosis, EIPA for macropinocytosis, filipin for caveolae‐mediated endocytosis, and cytochalasin D for phagocytosis (Figure 4B) [68, 69, 70, 71]. In both studies, Cy‐5 labeled FLuc mRNA was encapsulated in each **CORE** LNP and dosed onto DC 2.4 cells. Cy‐5 signal was then quantified by flow cytometry to determine the percentage of Cy‐5‐positive cells (Figure S15). To compare a head‐to‐head native cholesterol formulation against **C1‐6**, we utilized the SM‐102 LNP formulation which resembles the FDA‐approved Moderna COVID‐19 formulation (Figure S16). In analyzing this data several findings emerged: First, **C1:F13** had the highest percentage of uptake as indicated by Cy‐5 positive signal at both 2 and 24 h compared to all other formulations (Figure 4C). This finding also correlated to **C1:F13** having the highest FLuc expression seen in Figure 3E. Second, for both 2 and 24 h, the percent of cellular uptake decreased in succession between **C1, C2**, and **C3:F13** indicating that increasing ring size may decrease dendritic cell uptake (Figure 4C). Similarly, **C4:F13** had a higher cellular association compared to **C5:F13** which may suggest that the addition of the ethyl group (as compared to a methyl group) may decrease cellular uptake. Third, filipin had the highest percentage of cellular inhibition for all six **CORE** LNPs followed by cytochalasin D, suggesting both caveolae‐mediated endocytosis and phagocytosis play a role in the uptake pathway for each formulation (Figure 4D). Finally, SM‐102 LNP uptake was primarily inhibited via cytochalasin D suggesting phagocytosis is the primary uptake mechanism, and that cholesterol itself (and its modification) may play a role in the way DC 2.4 cells recognize LNPs as indicated by the statistically significant differences in uptake percentage and uptake mechanism between **CORE** LNPs and SM‐102 LNP.

**FIGURE 5:**
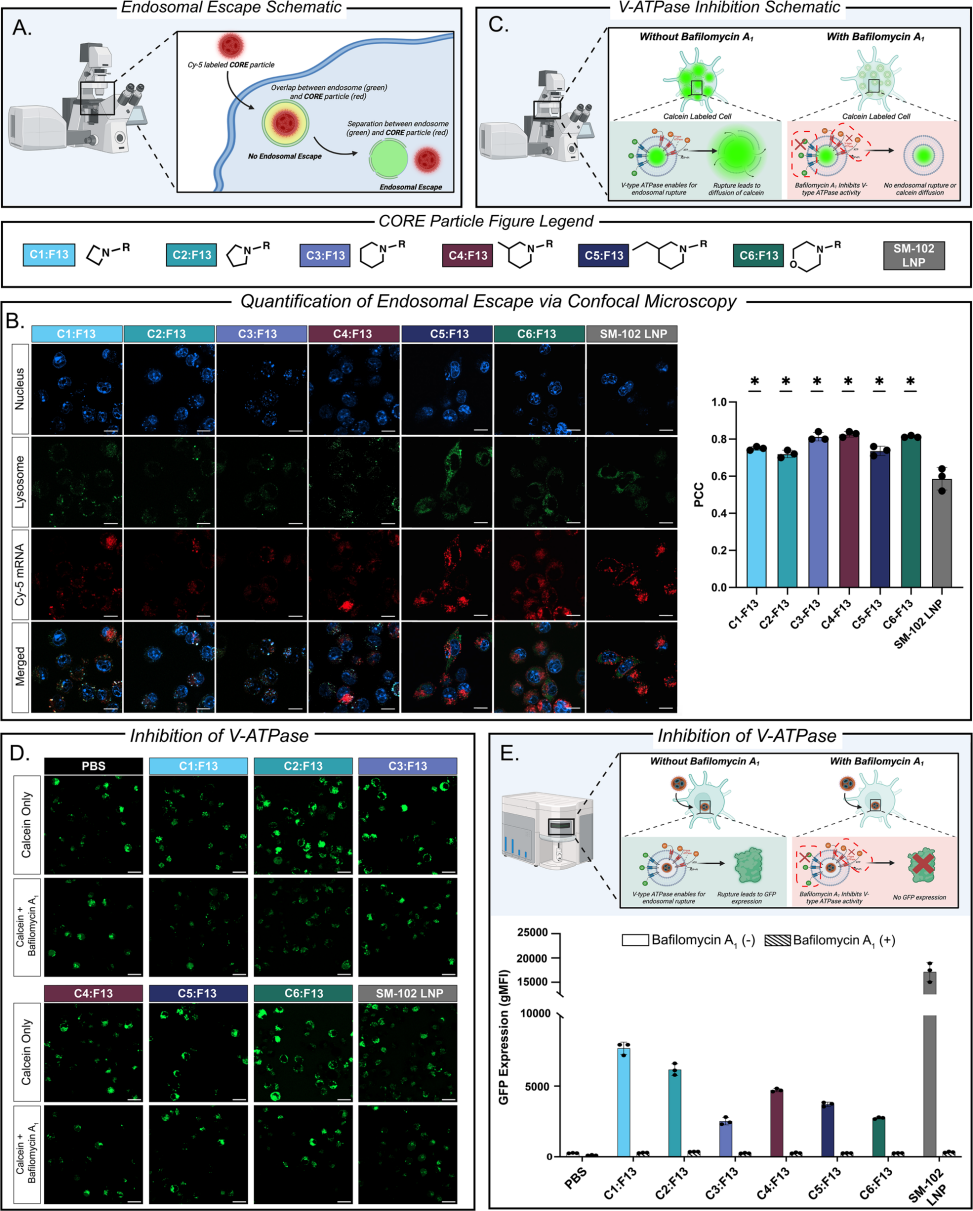
(a) Schematic representation of endosomal escape quantification via confocal microscopy. (b) Representative images and PCC quantification of **CORE** LNP taken via confocal microscopy where DC 2.4 cells were stained for nucleus (blue), lysosome (green) and dosed with **CORE** LNPs encapsulated with Cy‐5 FLuc mRNA (red). Scale bars 50 µm. (c) Schematic representation of calcein leakage assay evaluating the proton sponge effect on endosomal escape. (d) Representative confocal images of DC 2.4 cells treated with Calcein +/‐ bafilomycin A_1_ followed by **CORE** LNPs encapsulating FLuc mRNA. Scale bars 50 µm. (e) Quantification of geometric mean fluorescence intensity (gMFI) of GFP expression after treatment of **CORE** LNPs encapsulating GFP mRNA treated with or without bafilomycin A_1_. (All data represented as the mean ± standard deviation, *n* = 4, ^*^ *p*<0.05 as compared to SM‐102 LNP treatment group using one‐way ANOVA, Dunnett's test).

After studying the uptake pathway of lead **CORE** LNPs, we next sought to understand another important mechanistic step involved in mRNA LNP efficacy, namely endosomal escape, and the impact of the proton sponge effect on endosomal escape. Endosomal escape is an important bottleneck within the field given that many particles can be recycled out of cells upon cellular entry due to particles' inability to successfully rupture the endosome to escape into the cytosol. Given this important challenge, we sought to understand our lead **CORE** LNPs' endosomal escape properties [72, 73, 74]. To do so, we performed both a “label” and “label‐free” imaging approach to assess endosomal escape properties of each **CORE** LNP compared to our benchmark SM‐102 LNP. In our label‐based approach we encapsulated each **CORE** LNP with Cy‐5 labeled FLuc mRNA and dosed DC 2.4 cells. Cells were then stained with LysoTracker Green for imaging of the endosome and Hoechst 33342 DNA stain for imaging of the nucleus. Following staining, cells were imaged using confocal microscopy and endosomal escape of each formulation was quantified by calculating the Pearson's correlation coefficient (PCC) between the red Cy‐5 channel and the green endosome channel, where a PCC value closer to 1 indicates higher colocalization between the lysosome and mRNA thus suggesting lower endosomal escape (Figure 5A). Upon analyzing this labeled approach, we found two key findings: first, there was no significant difference in PCC across the six **CORE** LNPs, and second, it was found the SM‐102 LNP had a significantly lower PCC value as compared to each of the six **CORE** LNPs, suggesting the heterocyclic motifs combined with differences in formulation ratio may contribute to endosomal escape (Figure 5D).

To further complement our endosomal escape findings, we utilized a label‐free approach that does not rely on fluorescently labeled **CORE** LNPs. Instead, DC 2.4 cells were loaded with calcein—a green dye that remains trapped in endosomes unless they rupture. The cells were then incubated either with bafilomycin A_1_ – a proton pump inhibitor—or left untreated [75, 76]. After incubation, cells were dosed with **CORE** LNPs encapsulating FLuc mRNA and imaged using confocal microscopy. Here, calcein serves as a marker for endosomal integrity where it remains confined in the endosomes unless they rupture and release their contents into the cytosol. Bafilomycin A_1_‐treated cells (where endosomal rupture through the proton sponge effect is blocked) retain calcein in puncta within the intact endosomes, while untreated cells exhibit calcein spreading into the cytosol as the endosomes burst (Figure 5C). Upon imaging it was found all DC 2.4 cells treated with **CORE** LNPs, calcein and bafilomycin A_1_ had less calcein diffusion (indicated by dimmer cell images) as compared to **CORE** LNPs and calcein only. This may suggest an ATPase dependent endosomal escape pathway for **CORE** LNPs (Figure 5D).

Finally, to quantify the impact of endosomal escape—specifically the proton sponge effect—on mRNA expression, we utilized flow cytometry where DC 2.4 cells were pretreated with or without bafilomycin A_1_ and then dosed with **CORE** LNPs encapsulating GFP mRNA. GFP expression was then measured via flow cytometry, where reduced expression in bafilomycin A_1_‐treated cells suggests inhibition of endosomal escape indicating endosomal escape‐dependent mRNA expression (Figure S17). In analyzing this data several findings were uncovered. To begin, GFP expression was significantly decreased for all six **CORE** LNPs and SM‐102 LNP when co‐treated with bafilomycin A_1_, as indicated by a decrease in geometric mean fluorescence intensity (gMFI) across all groups when comparing with or without bafilomycin A_1_ treatment (Figure 5E). These findings suggest the ATPase pathway and the proton sponge effect contributes to the overall performance of **CORE** LNPs.

**FIGURE 6:**
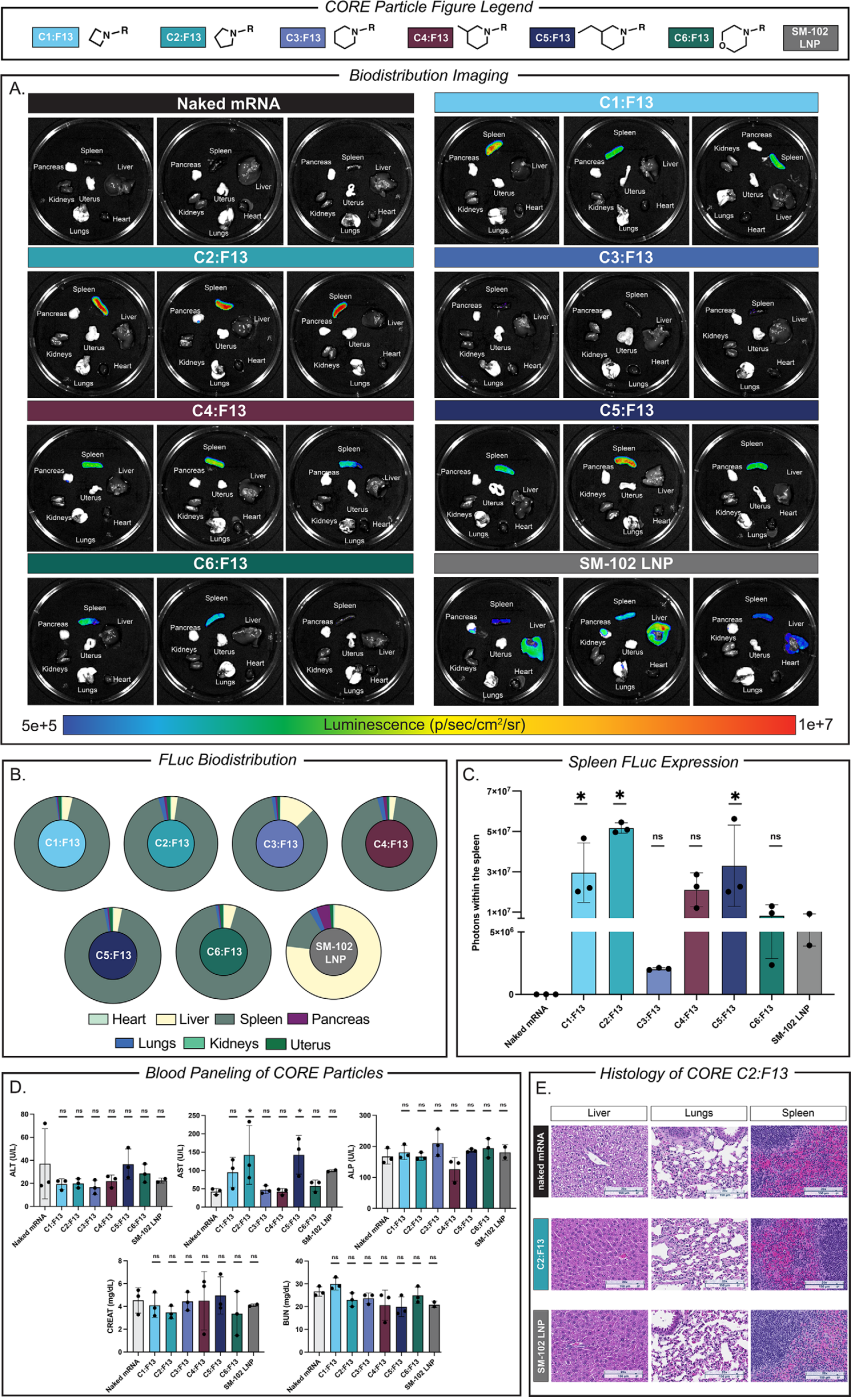
(A) Biodistribution imaging of lead **CORE** LNP formulations after IV administration. (B) Determination of organ biodistribution for lead **CORE** LNP formulations via FLuc expression. (C) Quantification of luminescence within the spleen of lead **CORE** LNP formulations. (D) ALT, AST, ALP, CREAT, and BUN quantification within the blood after 24 h treatment of lead **CORE** LNP formulations. (E) Histological evaluation of the liver, lung, and spleen after treatment with **C2:F13** compared to naked mRNA and SM‐102 LNP. (All data represented as the mean ± standard deviation, *n* = 3, ns *p*>0.05, ^*^ *p*<0.05 as compared to naked mRNA treatment group using one‐way ANOVA, Dunnett's test).

After evaluating our **CORE** LNP physical characteristics, performing formulation optimization to identify an optimal formulation for all six cholesterol derivatives, and studying the in vitro cellular mechanism of our lead **CORE** LNPs, we next sought to validate these particles in vivo for both mRNA biodistribution, cell type uptake, and safety/tolerability. To do so, we utilized C57BL/6 mice and injected our six lead **CORE** LNPs containing FLuc mRNA via intravenous injection. After 24 h, we evaluated FLuc protein expression of the liver, lung, spleen, kidneys, heart, pancreas, and ovaries/uterus via IVIS imaging. The highest performing **CORE** LNP (as determined by luminescence) was then labeled with DiD dye and administered IV into mice. After 24 h, spleens were digested and immune cells were quantified for DiD fluorescence via flow cytometry to quantify **CORE** LNP uptake within immune cells (Figure S19). The goal of this experiment was to further characterize in vivo performance by quantifying the percentage of splenic immune cells that internalized the lead **CORE** LNP **C2:F13** relative to a PBS‐treated control group. Additionally, we found it important to validate our **CORE** LNP safety profile for further translational application moving forward. Specifically, we evaluated weight loss 24 h after treatment and completed a full blood paneling measuring ALT, AST, ALP, Creatinine, BUN, and WBC count to fully evaluate the effects of each of our treatment groups. We chose these markers as they are important liver and kidney enzymes and elevated levels after administration may indicate signs of acute toxicity [77, 78]. Finally, we performed histological analysis on the liver, lung, and spleen on our highest performing **CORE** LNP formulation comparing to both SM‐102 LNP and naked mRNA. Upon IV injection and analysis of organs, several findings were uncovered. To begin, it was found all six lead **CORE** LNPs exhibited spleen specific expression after intravenous injection as shown by IVIS imaging of FLuc expression (Figure 6A). Specifically, it was found all six had greater than 95% mRNA expression within the spleen suggesting modifying cholesterol via its head group may alter biodistribution to the spleen irrespective of the identity of the head group motif. Additionally, SM‐102 LNP had 74% expression in the liver and 14% spleen expression further suggesting unmodified cholesterol may play a role in dictating delivery and expression to the liver after intravenous injection (Figure 6B). Furthermore, when looking at FLuc expression within the spleen it was found **C2:F13** had the highest average expression (quantified by luminescence) followed by **C5:F13** and **C1:F13** (Figure 6C). Interestingly it was found **C6:F13** and **C3:F13** had the lowest splenic expression suggesting that although the identity of cholesterol head group motifs may not affect overall biodistribution, they may influence the performance of **CORE** LNPs (Figure 6C). Of note, we also evaluated the biodistribution of a formulation consisting of the same molar ratio of **F13** but utilizing cholesterol rather than one of our six heterocyclic derivatives previously used (and we named this cholesterol formulation CH‐LNP, and the size, charge, and mRNA encapsulation efficiency of CH‐LNP can be found in Figure S18). Upon IV injection of CH‐LNP, IVIS imaging at 24 h showed biodistribution across both liver and spleen (whereas **CORE** LNPs with the same ratio but different cholesterol head groups showed expression in the spleen), suggesting that the chemistry may play a role in spleen‐selective expression (Figure S18). Additionally, splenic immune cell uptake studies showed two key findings. First, **C2:F13** was taken up by dendritic cells and T cells in the spleen at statistically significantly higher levels compared to PBS‐treated mice, and second **C2:F13** showed preferential DiD uptake of dendritic cells compared to immune cell subsets (B cells, Macrophages, and T cells) (Figure S19). Future studies will also utilize in vivo models to quantify mRNA expression to further understand delivery using **C2:F13**. Upon evaluating the safety and tolerability of our **CORE** LNPs it was found no mice had less than 5% weight loss 24 h after treatment (Figure S20) suggesting no acute toxicity after treatment with **CORE** LNPs. To further corroborate these tolerability findings, blood paneling analysis showed no significant increase in ALT, ALP, CREAT, and BUN for all **CORE** LNPs when compared to naked mRNA and the SM‐102 LNP (Figure 6D). AST levels were elevated for **C2:F13** and **C5:F13** as compared to naked mRNA, however, there was no significant difference in AST levels for either as compared to SM‐102 LNP (Figure 6D). Furthermore, histological analysis of the highest performing **CORE C2:F13** showed no significant histological damage in liver, lung, or spleen, further confirming the safety and tolerability of this platform (Figure 6E). Finally, WBC counts for all **CORE** LNPs were not significantly elevated as compared to naked mRNA or SM‐102 LNP (Figure S21).

## 3 Conclusion

In this paper, we developed an mRNA platform termed **C**h**O**lesterol alte**RE**d lipid nanoparticles (**CORE** LNPs), where we developed six novel heterocyclic cholesterol derivatives which were formulated into particles capable of delivering mRNA into dendritic cells in vitro and provided nearly 100% splenic expression in vivo. We specifically assessed their ability to deliver mRNA to the DC 2.4 dendritic cell line and identified the most effective **CORE** chemistries. We then studied the underlying mechanisms of cellular uptake and expression as well as the correlation between CORE structure, mechanism, and efficacy. Modulating the head group ring motif of the cholesterol derivative varied the particle characteristics and nanoparticle delivery uptake and endosomal escape mechanisms. Cholesterol derivative **C1** (azetidine head group) had the highest mRNA expression as indicated by FLuc and GFP expression in vitro. Interestingly, head group motifs correlated with percent of cellular uptake in vitro as an increase in the head size of each cholesterol derivative decreased cellular uptake in dendritic cells, which correlated to a decrease in mRNA expression. Upon in vivo validation of these particles, it was found all six lead **CORE** LNPs exhibited nearly 95% spleen expression after 24 h upon IV administration. Interestingly, **C2:F13** had the highest mRNA expression compared to other lead formulations, however all six formulations exhibited mRNA expression within the spleen. Going forward, further studies will be performed to further quantify dose‐normalizations and cell‐type preference. Specifically, **CORE** particles encapsulating mRNAs encoding for fluorescent proteins and radiolabeling studies will be performed to better understand additional performance aspects of these systems. Further studies will also investigate repeat dosing of **CORE** LNPs and include an “empty” **CORE** LNP as an additional treatment group. Future research will expand on evaluating **CORE** chemistries and investigating their in vitro and in vivo immune response. Additionally, comparing our **CORE** platform to other mRNA delivery platforms in the field which have shown splenic delivery will be important, including SORT particles and commercially available cholesterol derivatives, to further validate the utility of developing novel cholesterol derivatives for addressing extrahepatic mRNA delivery [23, 24, 79, 80, 81, 82, 83]. Notably, in this work we present **CORE** as one potential platform for splenic mRNA delivery in the context of others and show that our heterocyclic cholesterol derivatives may be used as one potential strategy to achieve spleen expression in vivo [23, 24, 62, 84]. We hope that our development of a novel cholesterol chemistry system for mRNA delivery systems will inform the next generation of vaccine development, with a perhaps longer‐term goal of further advancing health‐based strategies for the study and prevention of disease.

## 4 Experimental Section

### 4.1 Materials

*para*‐Toluenesulfonic acid, Azetidine, Pyrrolidine, Piperidine, 3‐Methylpiperidine, and Morpholine, Cholesterol, Propiolic Acid, Dulbecco's phosphate‐buffered saline (DPBS), phosphate‐buffered saline (PBS, pH *7.4*), sodium citrate, citric acid, penicillin‐streptomycin, Triton X‐100, filipin from *S*. *filipinensis*, cytochalasin D, (N‐ethyl‐N‐isopropyl) amiloride (EIPA), Tween 20, DC 2.4 cells (Cat. # SCC142) were all purchased from Sigma‐Aldrich. 3‐Ethylpiperidine was purchased from Santa Cruz Biotechnologies. 1,2‐dioleoyl‐*sn*‐glycero‐3‐phosphoethanolamine (DOPE), 1,2‐dimyristoyl‐*sn*‐glycero‐3‐phosphoethanolamine‐N‐[methoxy‐(polyethyleneglycol)‐1000] (14:0 PEG 1K), 1,2‐dimyristoyl‐rac‐glycero‐3‐methoxypolyethylene glycol‐2000 (DMG‐PEG 2000), and 1,2‐distearoyl‐sn‐glycero‐3‐phosphocholine (DSPC) were purchased from Avanti. The ionizable lipid SM‐102 was bought from Broadpharm. RPMI 1640, Fetal Bovine Serum (FBS), LysoTracker Green, Hoechst 33342, were purchased from Life Technologies. CleanCap Firefly luciferase (FLuc) mRNA (5‐moU) was purchased from Hongene Biotech. EZ Cap Cy‐5 labeled FLuc mRNA was purchased from APEXBio. Quant‐iT RiboGreen RNA assay kit and Alamar Blue Cell Viability Reagent was purchased from Thermo Fisher Scientific, USA. Bright‐Glo Luciferase assay system was obtained from Promega. Pitstop 2 was purchased from Abcam. Jurkat cells, Clone E6‐1 (ATCC TIB‐152) were purchased from American Type Culture Collection (ATCC). Ultrapure water (Milli‐Q) with a resistivity greater than 18.2 MΩ·cm was used in all experiments and obtained from a three stage Millipore Milli‐Q Plus 185 purification system. All chemicals were used without further purification.

### 4.2 Synthesis of C1‐C6

Five grams of Cholesterol (12.93 mmol, 1 eq, Sigma–Aldrich C8667) was added to a reaction flask containing 125 mL of Toluene. 2.42 mL of Propiolic Acid (32.33 mmol, 2.5 equivalents Sigma–Aldrich P51400) was added to the reaction, immediately followed by 222 mg of *para*‐Toluenesulfonic Acid (1.29 mmol, 0.1 equivalents Sigma–Aldrich 8.14725). The reaction was heated under reflux at 165°C using a Dean‐Stark apparatus for 24 h. The reaction was later cooled to room temperature, and the solvent was removed using rotary evaporation. The crude product was then purified through a silica column with a gradient elution profile comprising hexane and diethyl ether (100:0 —> 96:4 —> 94:6) as the mobile phase to afford an intermediate compound with a yield of 74%. After purification, either Azetidine (5.86 mg, 0.103 mmol, 1.0 equivalents, Sigma–Aldrich 281069), Pyrrolidine (7.28 mg, 0.103 mmol, 1.0 equivalents, Sigma–Aldrich P73803), Piperidine (8.72 mg, 0.103 mmol, 1.0 equivalents, Sigma–Aldrich 104094) 3‐Methylpiperidine (10.17 mg, 0.103 mmol, 1.0 equivalents, Sigma–Aldrich M73001) 3‐Ethylpiperidine (11.59 mg, 0.103 mmol, 1.0 equivalents, Santa Cruz Biotechnologies sc‐346905), or Morpholine (8.92 mg, 0.103 mmol, 1.0 equivalents, 134236 Sigma–Aldrich) was added into 0.5 mL of Chloroform and left to stir. A 0.5 mL solution of Chloroform containing the intermediate (45 mg, (0.103 mmol, 1.0 equivalents) was then added into the reaction flask and left to stir overnight at 45°C. The solvent was then removed via rotary evaporation to yield **C1‐C6** respectively.

### 4.3 Design of Experiment Formulation Generation

Formulation ratios were generated using design of experiment via JMP 17 software. Specifically, a 3^4^ × 2^1^ definitive screening design was employed using 3 three‐level quantitative factors (SM‐102 molar %, **CX** molar %, phospholipid molar %, and PEG mol %) and 1 two‐level qualitative factor (DOPE/DSPC). The range of molar percentage factors were determined based on the SM‐102 LNP formulation ratio (35%–70% ionizable lipid, 20%–55% respective cholesterol derivative, 3%–21% phospholipid, and 0.4%–2.5% PEG‐lipid). 13 formulations were generated from this design and were subsequently tested for each cholesterol derivative.

### 4.4 Formulation **CORE** LNPs

An ethanol phase was prepared by combining the ionizable lipid SM‐102, dioleoyl‐sn‐glycero‐3‐phosphoethanolamine, **C1, C2, C3, C4, C5,** or **C6**, 1,2‐dimyristoyl‐sn‐glycero‐3, and 1,2‐dimyristoyl‐sn‐glycero‐3‐phosphoethanolamine‐N‐[methoxy‐(polyethyleneglycol)] (MW 1000). The aqueous phase was generated by mixing FLuc/Cy‐5 labeled FLuc mRNA (0.1 mg mL^−^^1^) in 10 mm citrate buffer (pH *3*). These two phases were then combined utilizing a syringe pump (Harvard Apparatus Pump 33 DDS) at a 3:1 aqueous‐to‐ethanol ratio with a flow rate of 900 and 300 uL/min using a herringbone mixer microfluidic chip device (LabSmith). The **CORE** LNPs were then dialyzed against 1X PBS using a 20 kDa MWCO cassette at 4°C for 2 h in a cold room and subsequently stored at 4°C until further use. For **CORE** LNPs prepared for CryoTEM, particles were dialyzed against 0.1X PBS and 3 µL of the **C2:F13** loaded onto a lacey copper grid coated with a continuous carbon film. The grid was frozen and then imaged using a Talos Arctica electron microscope.

### 4.5 Characterization of **CORE** LNPs

All **CORE** LNP formulations were analyzed using dynamic light scattering (DLS) on a NanoBrook 90 Plus Zeta instrument (Brookhaven, USA). For DLS and zeta potential measurements, LNPs were diluted in 1X PBS (pH *7.4*) and 0.1X PBS (pH 7.4), respectively. Encapsulation efficiency of FLuc/Cy‐5 labeled FLuc/GFP mRNA was determined using Quant‐iT RiboGreen RNA assay kit and measured with a SpectraMax microplate reader (Molecular Devices, USA) at an excitation wavelength of 480 nm and an emission wavelength of 520 nm. To assess total mRNA content, **CORE** LNPs were completely solubilized using 1X Triton X‐100.

### 4.6 In Vitro Dose Response of FLuc Expression

DC 2.4 cells and Jurkat Cells were plated at a density of 1 × 10^4^ cells per well in 96‐well plates and incubated at 37 °C. The following day, the culture media was removed, and 100 µL of either 500 ng/mL FLuc mRNA–encapsulated **CORE** LNPs, naked FLuc mRNA, or PBS (negative control) was added to the cells. The cells were then incubated for 24 h. After incubation, 100 µL of Bright‐Glo Luciferase assay buffer was added to each well, and luminescence was measured using the luminescence function of a SpectraMax microplate reader. Luminescence values were normalized to the percentage of viable cells in each treatment group to calculate luminescence per live cell.

### 4.7 Cell Viability by Alamar Blue Cell Viability Reagent

DC 2.4 and Jurkat cells were cultured in RPMI supplemented with 10% fetal bovine serum (FBS) and 1% penicillin‐streptomycin at 37 °C in a 5% CO_2_ incubator. Respective Cells were seeded in 96‐well plates at a density of 1 × 10^4^ cells per well in 100 µL of media and incubated overnight. The following day, the media was removed, and cells were treated with 100 µL of fresh media (cell control), 100 µL of ethanol in RPMI (negative “kill” control), or 100 µL of **CORE** LNPs or naked FLuc mRNA at 500 ng/mL mRNA concentration for 24 h. After treatment, a 10% Alamar Blue reagent solution was prepared. Spent media was aspirated and replaced with 100 µL of Alamar Blue solution, and cells were incubated for 3 h. Absorbance was then measured at 570 nm with a 600 nm reference wavelength using a SpectraMax microplate reader. Untreated cells containing only RPMI 1640 media served as background controls. Cell viability was calculated by normalizing the absorbance of each treatment group to that of the untreated cells.

### 4.8 Cellular Uptake

DC 2.4 cells were seeded in a transparent, treated 24‐well plate at a cell density of 5 × 10^4^ cells per well and incubated in RPMI media at 37 °C for 24 h. Cells were then treated with 400 µL of 500 ng/mL Cy‐5 labeled FLuc mRNA encapsulated in **CORE** LNPs or SM‐102 LNPs and incubated for the designated time points (2 and 24 h) at 37 °C. At each time point, cells were washed three times with DPBS and detached using 0.5% trypsin‐EDTA. The samples were diluted in RPMI, centrifuged at 300 × g, the supernatant was removed, and cells were resuspended in 300 µL of PBS. Samples were analyzed using an Attune Flow Cytometer (Thermo Fisher Scientific, USA), and cellular uptake was quantified by gating for cells exhibiting higher Cy‐5 fluorescence intensity compared to untreated control cells.

### 4.9 Cellular Uptake Mechanism

DC 2.4 cells were seeded in a transparent 24‐well plate at a density of 8 × 10^4^ cells per well and incubated in complete RPMI media at 37 °C for 24 h. Cells were then treated with endocytosis inhibitors—pitstop 2, EIPA, filipin from *S. filipinensis*, and cytochalasin D—at final concentrations of 10, 30, 10 µg/mL, 33.4 mm, and 40 µm, respectively. After 15 min of pre‐incubation with the inhibitors, 300 µL of 500 ng/mL **CORE** LNPs or SM‐102 LNP encapsulating Cy‐5 labeled FLuc mRNA was added, and cells were incubated for 2 h at 37 °C. Following incubation, media was removed, and cells were washed three times with DPBS and detached using a cell scraper. Samples were diluted in RPMI, centrifuged, the supernatant removed, and resuspended in 300 µL of 1X PBS. Cellular uptake was analyzed using an Attune Flow Cytometer (Thermo Fisher Scientific, USA), and the extent of inhibition (%) was calculated by normalizing Cy‐5 fluorescence intensity of the inhibitor‐treated groups to that of uninhibited controls.

### 4.10 Imaging of V‐ATPase Inhibition

DC 2.4 cells were seeded at a density of 5 × 10^4^ cells per well in an eight well coverslip µ‐slide (Ibidi) at 37°C overnight. The next day, the culture media was replaced with 150 µL of media or media containing bafilomycin A_1_ (200 nm). Subsequently, calcein (20 µL, 150 µg/mL) was added to each well on top of existing media. **CORE** LNPs and SM‐102 LNP formulations encapsulating FLuc mRNA were then treated to respective wells for 4 h at a mRNA concentration of 500 ng/mL. Samples were then washed four times with DPBS. Finally, cells were imaged and captured by CLSM (Zeiss SPX980) at 40x oil immersion. All images were processed using WCIF ImageJ software.

### 4.11 Quantification of GFP Expression after V‐ATPase Inhibition

DC 2.4 cells were seeded at a density of 5 × 10^4^ cells per well in an eight well coverslip µ‐slide (Ibidi) at 37°C overnight. The next day, the culture media was replaced with 150 µL of media or media containing bafilomycin A_1_ (200 nm). After 15 min of incubation with bafilomycin A_1_, **CORE** LNPs and SM‐102 LNP was added into respective wells and incubated for 24 h. The culture medium was then removed, and cells were washed three times with DPBS and detached with 0.5% trypsin‐EDTA. The cells were then analyzed via Attune Flow Cytometer (Thermo Fisher Scientific, USA).

### 4.12 Endosomal Escape of **CORE** LNPs

DC 2.4 cells were seeded at a density of 8 × 10^4^ cells per well in µ‐slide 8‐well coverslip slides and incubated overnight at 37 °C. The following day, media was aspirated, and 300 µL of **CORE** LNPs or SM‐102 LNP encapsulating Cy‐5 labeled FLuc mRNA (500 ng/mL mRNA) was added to each well and incubated for 2 h at 37 °C. Cells were then washed three times with DPBS, and 300 µL of 100 nm LysoTracker Green in prewarmed culture media was added for a 2‐h incubation. Afterward, cells were washed three times with DPBS, and nuclei were stained with 1 µg/mL Hoechst 33342 for 15 min. Live‐cell imaging was performed using a confocal laser scanning microscope (CLSM, Zeiss SPX980) with a 40x oil immersion objective. Images (control images and all treatment groups) were processed using WCIF ImageJ software. Experiments were conducted in triplicate, and five representative images (>50 cells per image) were analyzed to calculate the Pearson Correlation Coefficient (PCC).

### 4.13 Animal Studies of FLuc mRNA Encapsulated **CORE** LNPs

All animal studies were approved by the UNC Institutional Animal Care and Use Committee and conducted in compliance with applicable local, state, and federal regulations, supported by the UNC Lineberger Animal Services Core at the University of North Carolina at Chapel Hill. FLuc mRNA–encapsulated **CORE** LNPs, SM‐102, and CH‐LNPs were prepared, with FLuc mRNA alone serving as a positive control and PBS as a negative control. Female C57BL/6 mice (Jackson Laboratory, 18–21 g, 6 weeks) were housed in a ventilated hood with a wood shaving bedding and provided food and water throughout the studies. Each mice were injected via the tail vein with each formulation at a dose of 0.65 mg/kg. After 24 h, mice received an intraperitoneal injection of 130 µL D‐luciferin (30 mg/mL in PBS). Fifteen minutes later, mice were euthanized, and organs (pancreas, spleen, liver, kidneys, ovaries, lung, and heart) were harvested and imaged using an In Vivo Imaging System (IVIS, PerkinElmer, Waltham, MA). Luminescence was quantified using AuRA software (Spectral Instruments Imaging). Biodistribution of each organ was calculated by dividing the luminescence value of respective organ by the sum of all organ luminescence and multiplied by 100.

### 4.14 In Vivo Blood Paneling Studies

Twenty‐four hours post‐dosing, 250 µL of blood was collected from each mice via cardiac puncture. Of this blood, 60 µL was transferred into K_2_EDTA microcontainers (BD), while the remaining 190 µL was collected into Minicollect tubes (BD) and kept at room temperature. Serum was separated by centrifugation at 1300 rcf for 10 min. Complete blood count analyses, as well as assessments of liver and kidney function, were then performed on the collected samples.

### 4.15 Preparation of Histology of Organs

For H&E staining, the liver, spleen, and lung were fixed in 10% neutral buffered formalin, processed routinely into paraffin, and sectioned at 5 µm onto positively charged slides. Sections were baked at 60 °C for at least 1 h, deparaffinized in xylene, and hydrated through graded ethanol series before hematoxylin and eosin (H&E) staining. Staining was performed using a Leica Biosystems Autostainer XL with Hematoxylin (Richard‐Allen Scientific, 7211) for 2 min and Eosin‐Y (Richard‐Allen Scientific, 7111) for 1 min. Clarifier 2 (7402) and Bluing (7111) solutions (Richard‐Allen Scientific) were used to differentiate the reaction. Following staining, slides were dehydrated and coverslipped with Cytoseal 60 (Thermo Fisher Scientific, 8310–4), and histological analysis was performed on each sample.

### 4.16 Statistical Analysis

All statistical analyses were calculated using GraphPad Prism (GraphPad Software, CA, USA). Analysis of Variance (ANOVA) was employed to assess differences among multiple groups. Dunnett's multiple comparison test was used for multiple comparison testing. p‐value where *p* > 0.05 was considered not statistically significant and “*” indicates a statistically significant difference between groups where *p* < 0.05.

## Author Contributions

**Eshan A. Narasipura**: Conceptualization, Investigation, Formal analysis, Writing – Original Draft, Visualization. **Vincent Fung**: Investigation, Formal analysis, Writing – Review and Editing. **Rachel VanKeulen‐Miller**: Writing – Review and Editing, Visualization. **Palas Balakdas Tiwade**: Methodology. **Owen S. Fenton**: Conceptualization, Supervision, Writing – Review and Editing, Funding Acquisition.

## Conflicts of Interest

The authors declare no conflicts of interest.

## Declaration of Generative AI Usage

The generative AI tools Perplexity and Chat GPT were utilized solely for the grammatical editing and proofing of this manuscript.

## Supporting information




**Supporting File 1**: adhm71011‐sup‐0001‐SuppMat.pdf.

**Supporting File 2**: adhm71011‐sup‐0002‐DataFile.xlsx.

## Data Availability

The data that support the findings of this study are available from the corresponding author upon reasonable request.
