# Supplementary material for: CORE: Cholesterol Altered Lipid Nanoparticles for Splenic Expression of mRNA Payloads
Source: Adv Healthc Mater. 2026 Mar 28;15(19):e05862. doi: 10.1002/adhm.202505862 (PMC13206406; doi:10.1002/adhm.202505862)
Supplement: Supplementary file 1 — Supporting File 1: adhm71011‐sup‐0001‐SuppMat.pdf. [file ADHM-15-0-s001.pdf]

CORE: Cholesterol Altered Lipid Nanoparticles for Splenic Expression of mRNA Payloads

Eshan A. Narasipura [a], *Vincent Fung* [a], *Rachel VanKeulen-Miller* [b], *Palas B. Tiwade* [a], *Owen S. Fenton* [a,b*]

[a]-Division of Pharmacoengineering and Molecular Pharmaceutics, Eshelman School of Pharmacy, University of North Carolina at Chapel Hill, Chapel Hill, NC 27599, USA

[b]-Department of Pharmacology, School of Medicine, University of North Carolina at Chapel Hill, Chapel Hill, NC, 27599, USA

*E-mail: osfenton@unc.edu

Supplementary Data

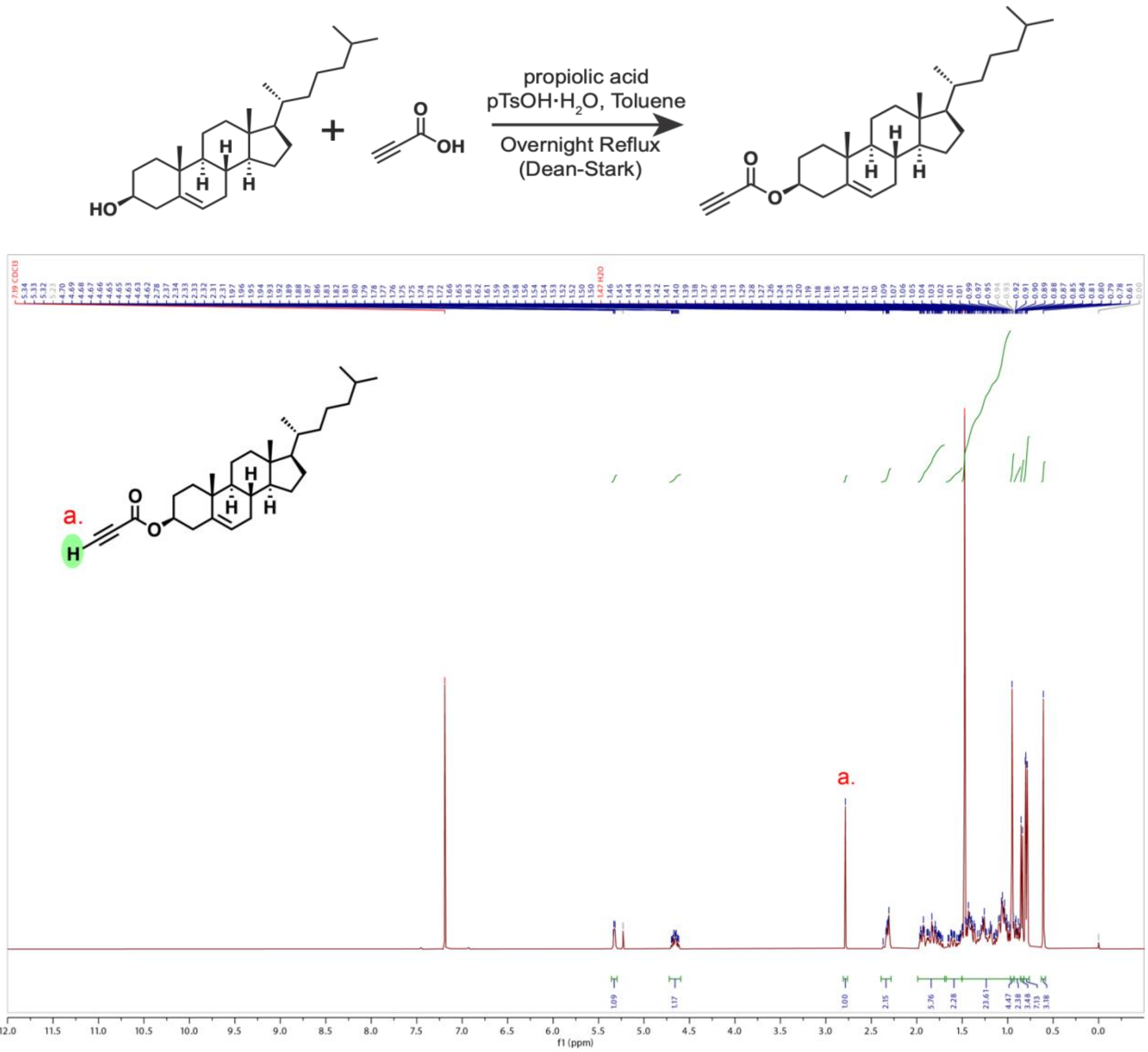

Supplementary Figure 1: Reaction Scheme and ^{1}H NMR of intermediate common core compound.

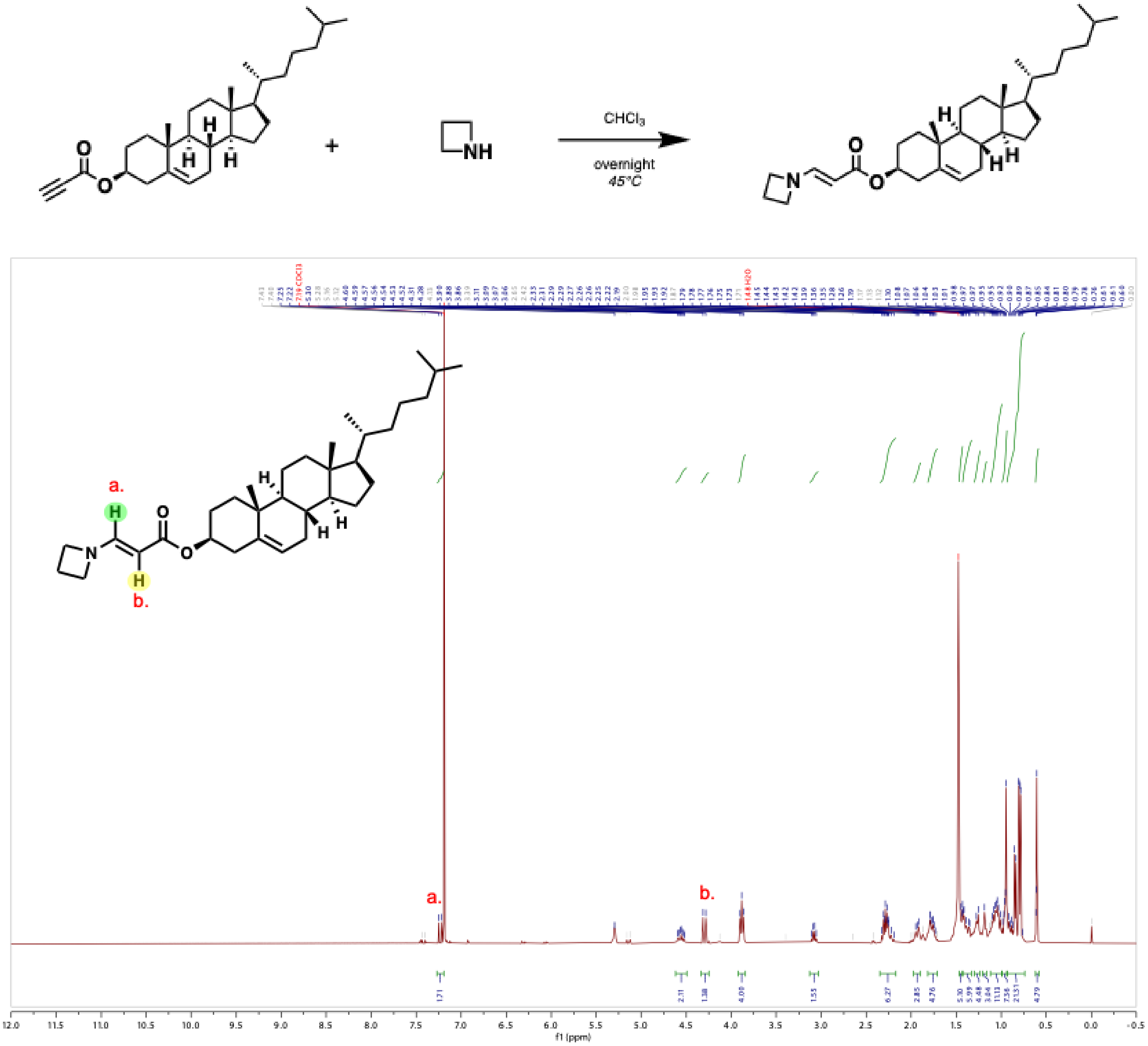

Supplementary Figure 2: Reaction Scheme and ^{1}H NMR of **C1**.

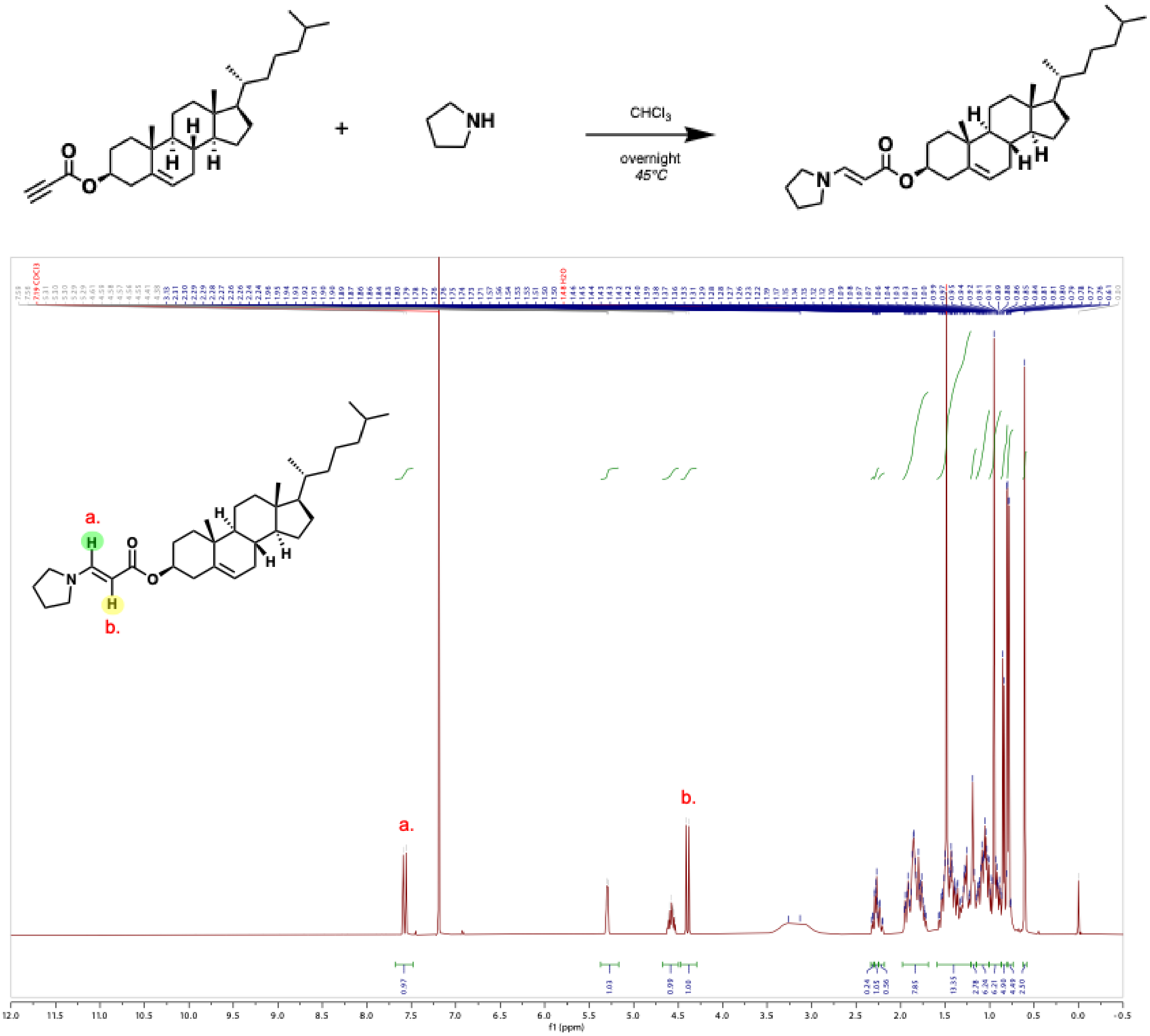

Supplementary Figure 3: Reaction Scheme and ^{1}H NMR of **C2**.

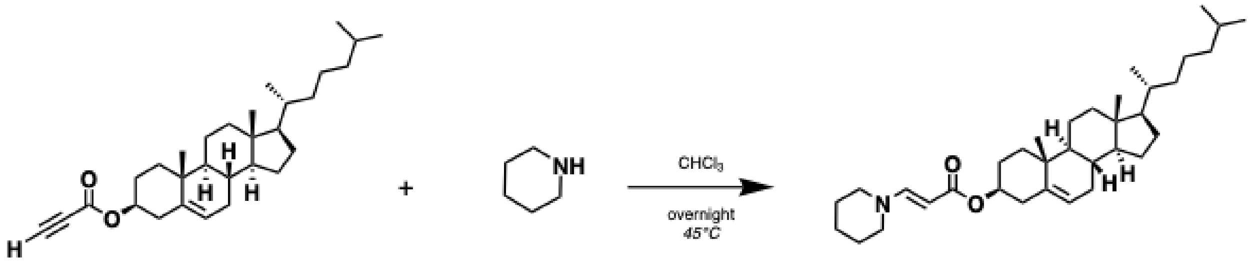

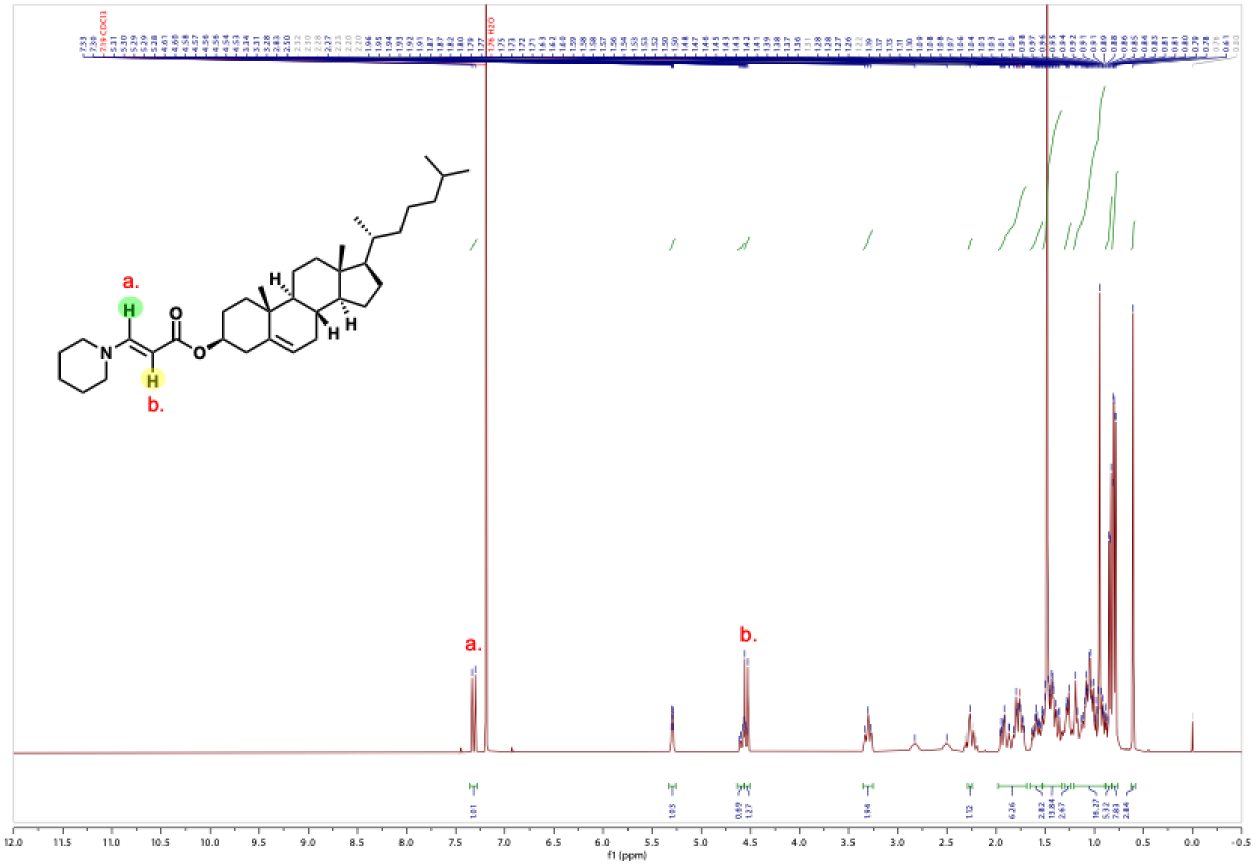

Supplementary Figure 4: Reaction Scheme and ^{1}H NMR of **C3**.

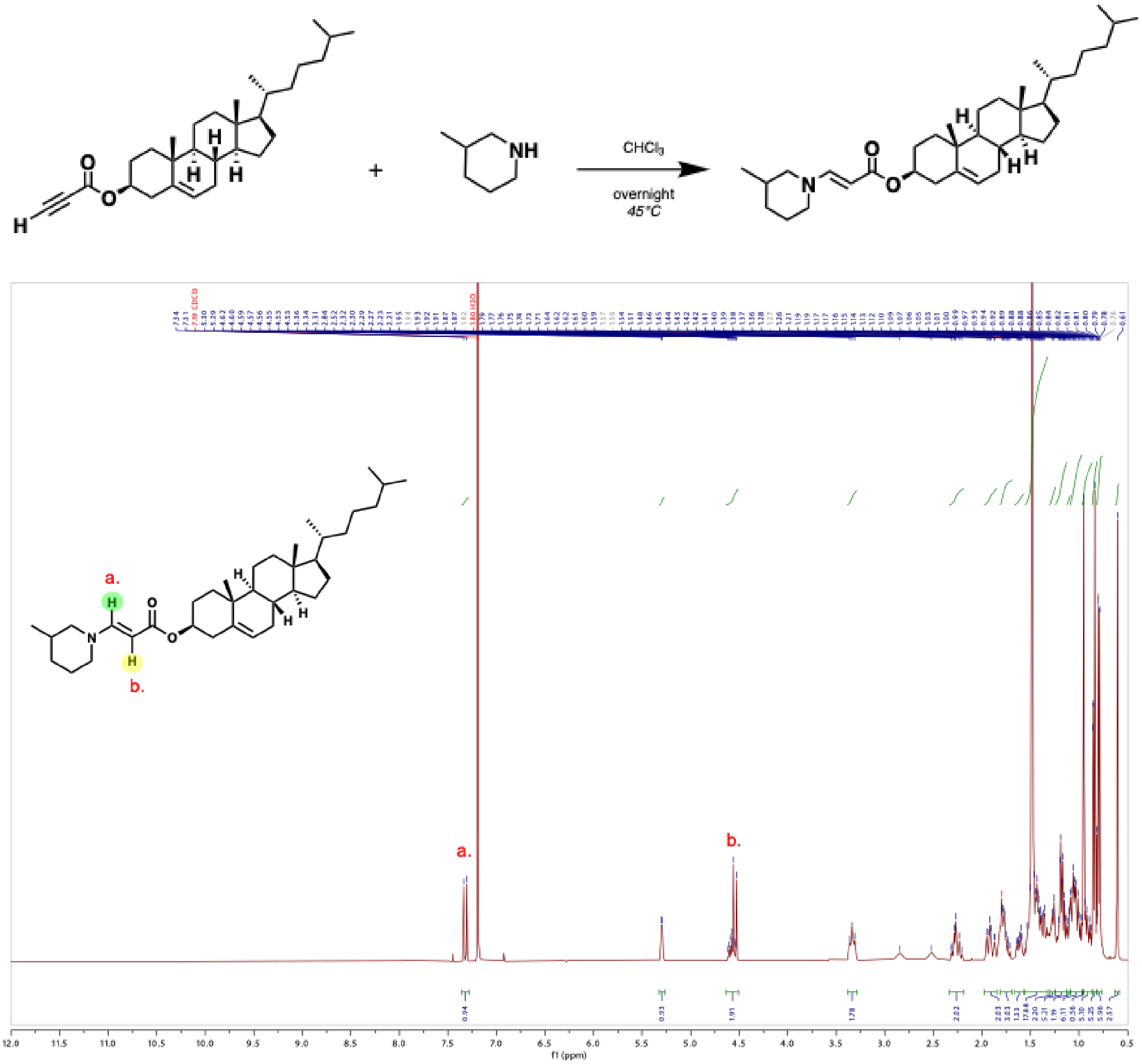

Supplementary Figure 5: Reaction Scheme and ^{1}H NMR of **C4**.

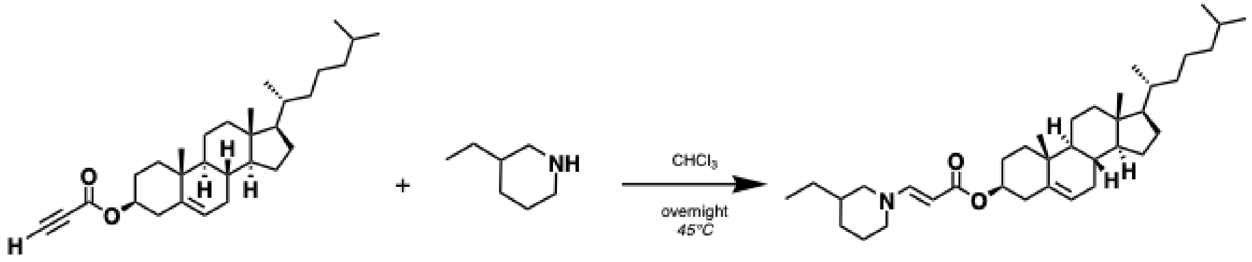

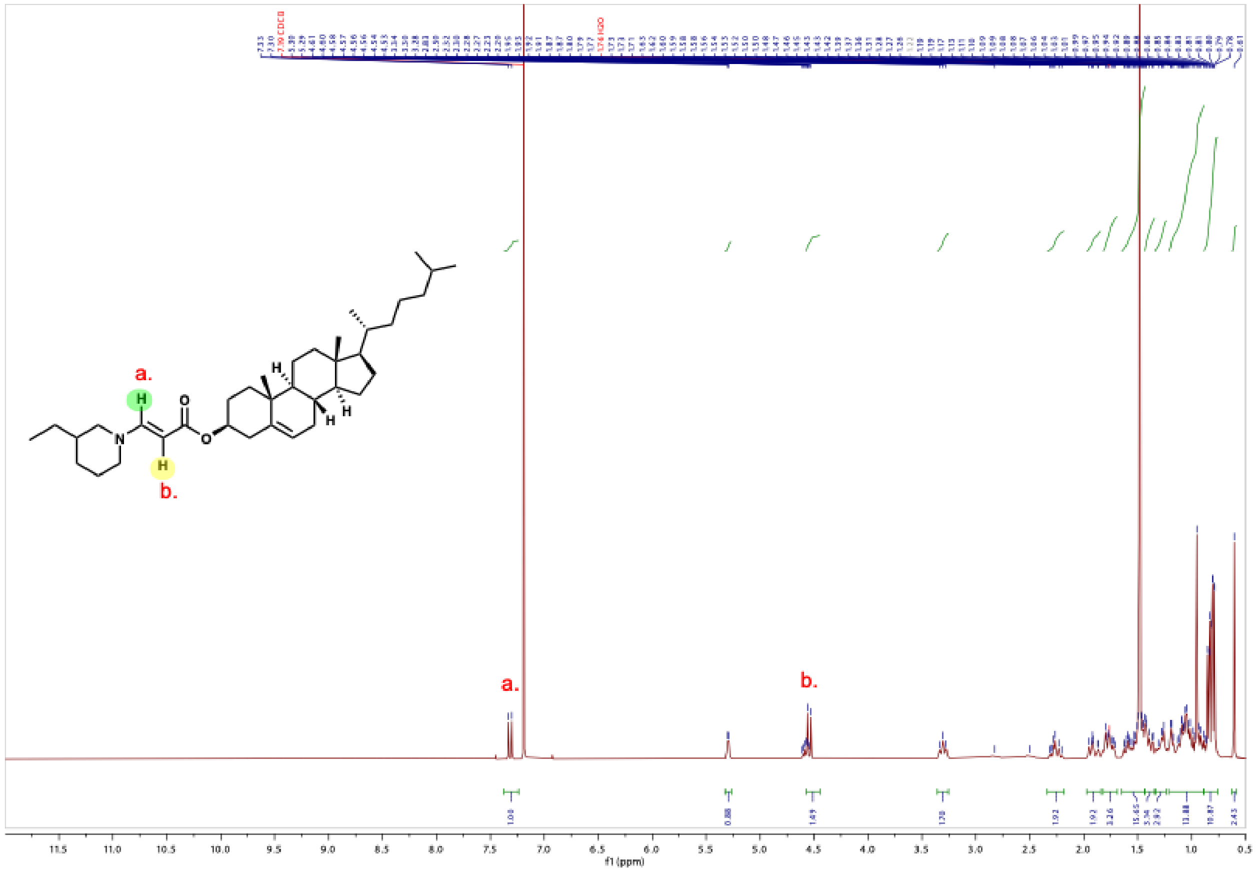

Supplementary Figure 6: Reaction Scheme and ^{1}H NMR of **C5**.

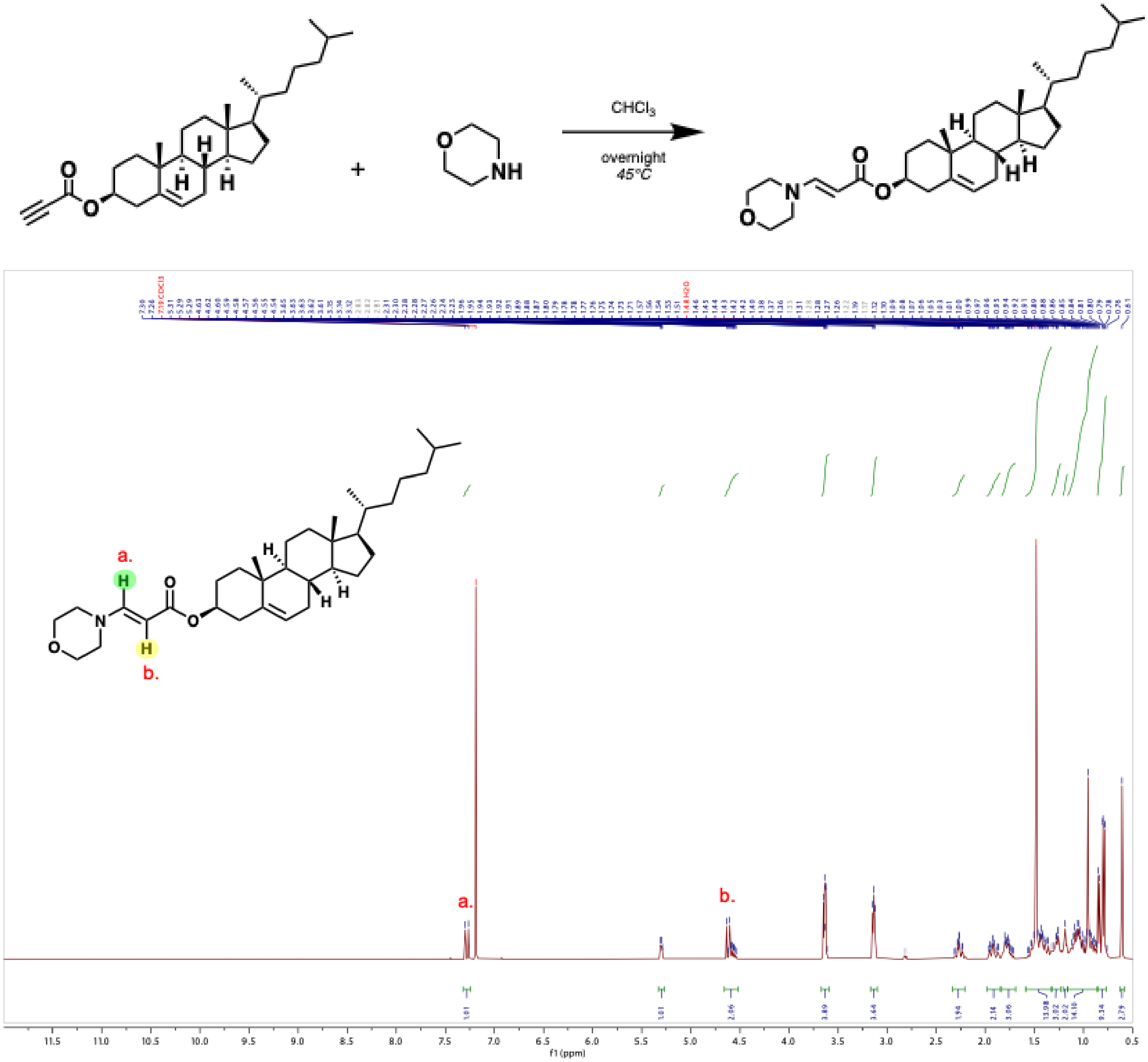

Supplementary Figure 7: Reaction Scheme and ^{1}H NMR of **C6**.

Physicochemical Parameter Measured	Cholesterol	C1	C2	C3	C4	C5	C6
pH at 5.5	0	1	1	1	1	1	1
logP	7.1	8	8.5	8.98	9.3	9.79	7.9
logS	-9.5	-10.89	-10.87	-11	-11.5	-11.9	-10.58
Maximum Projection Radius	10.21	11.29	11.87	11.46	11.68	11.69	12.28
Rotatable Bond Count	5	9	9	9	9	10	9
Ring Count	4	5	5	5	5	5	5
HLB	1.22	5.11	4.77	4.44	4.11	3.78	6.2
Hydrogen Bond Donor Count	1	0	0	0	0	0	0
Hydrogen Bond Acceptor Count	1	2	2	2	2	2	3
Topological Polar Surface Area	20.23	29.54	29.54	29.54	29.54	29.54	38.77
Min Projection Radius	4.69	6.62	6.65	7.24	6.2	6.49	5.58
Solvent Accessible Surface Area	599.74	786.18	798	814.6	842	875	813
Minimum Projection Area	46.61	72.95	74.53	82.1	86.6	90.27	64.75
Max Projection Area	122.26	148.38	153.55	154	161.8	167	157.22
Atom Count	74	89	92	95	98	101	93
Van der Waals Volume	428.03	525.8	543.89	560	578	594	554
Van der Waals Area	722.3	877.7	905	934.8	964	996	919.8
Heavy Atom Count	28	36	37	38	39	40	38
Polarizability	47.98	59	61.16	63	64.85	66.69	61.8
Molar Refractivity	120.62	151	155.66	160.26	164.73	169.33	157.2
Asymmetrical Atom Count	8	8	8	8	9	9	8
Hetero Ring Count	0	1	1	1	1	1	1
Charge at pH 7.5	0	0.56	0.91	0.5	0.67	0.75	0.57

Supplementary Figure 8: Values of each physicochemical property measured for Cholesterol and **C1-6** (all parameters measured using ChemAxon software).

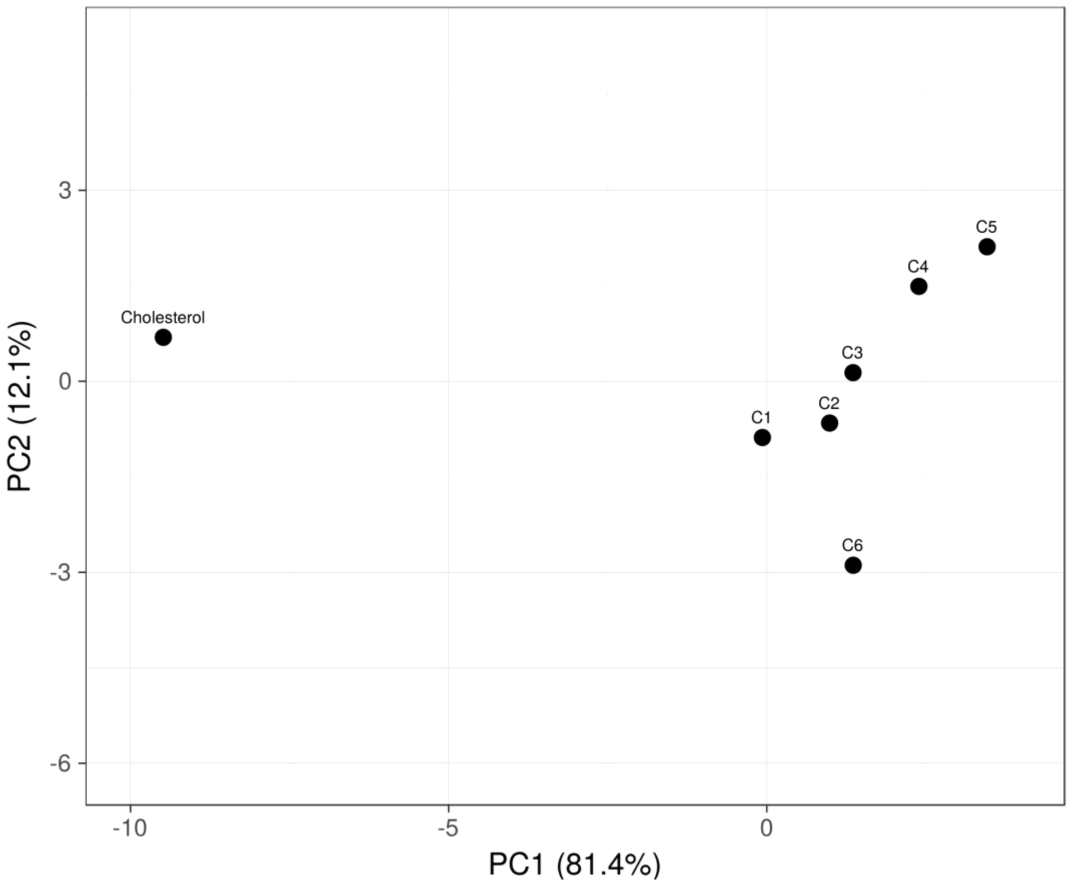

Supplementary Figure 9: PCA Plot visualizing the 23 physicochemical parameters measured for cholesterol and **C1-6**.

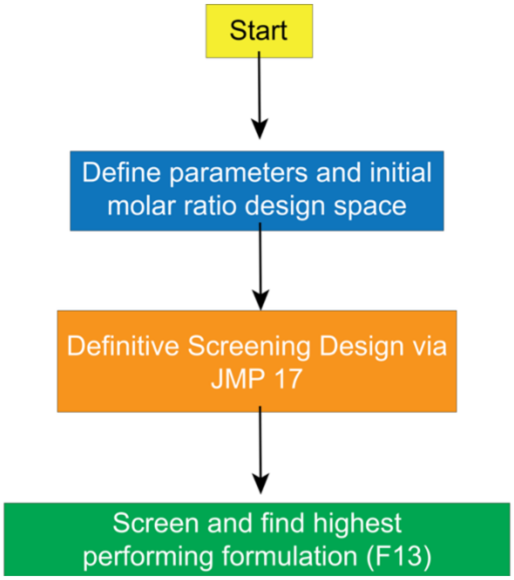

Supplementary Figure 10: Schematic workflow utilized for design of experiment-based formulation generation and screening of the **CORE** LNPs.

Formulation Name	SM-102	Heterocyclic Cholesterol Derivative	Phospholipid	Phospholipid Type	14:0 PEG 1K
**F1**	69	24.5	4.5	DOPE	2
**F2**	50	46	3.6	DOPE	0.4
**F3**	50	27	20	DSPC	3
**F4**	50	39	9.5	DSPC	1.5
**F5**	50	39	9.5	DOPE	1.5
**F6**	51	33	14	DOPE	2
**F7**	45	42.5	12	DSPC	0.5
**F8**	64	23	10.5	DSPC	2.5
**F9**	48	46	5	DSPC	1
**F10**	38.5	52	9	DOPE	0.5
**F11**	40	45	13	DOPE	2
**F12**	61	22	16	DOPE	1
**F13**	51	28	20.5	DOPE	0.5

Supplementary Figure 11: Formulation ratios used for evaluating all **CORE** formulations (lead formulation **F13** highlighted in yellow).

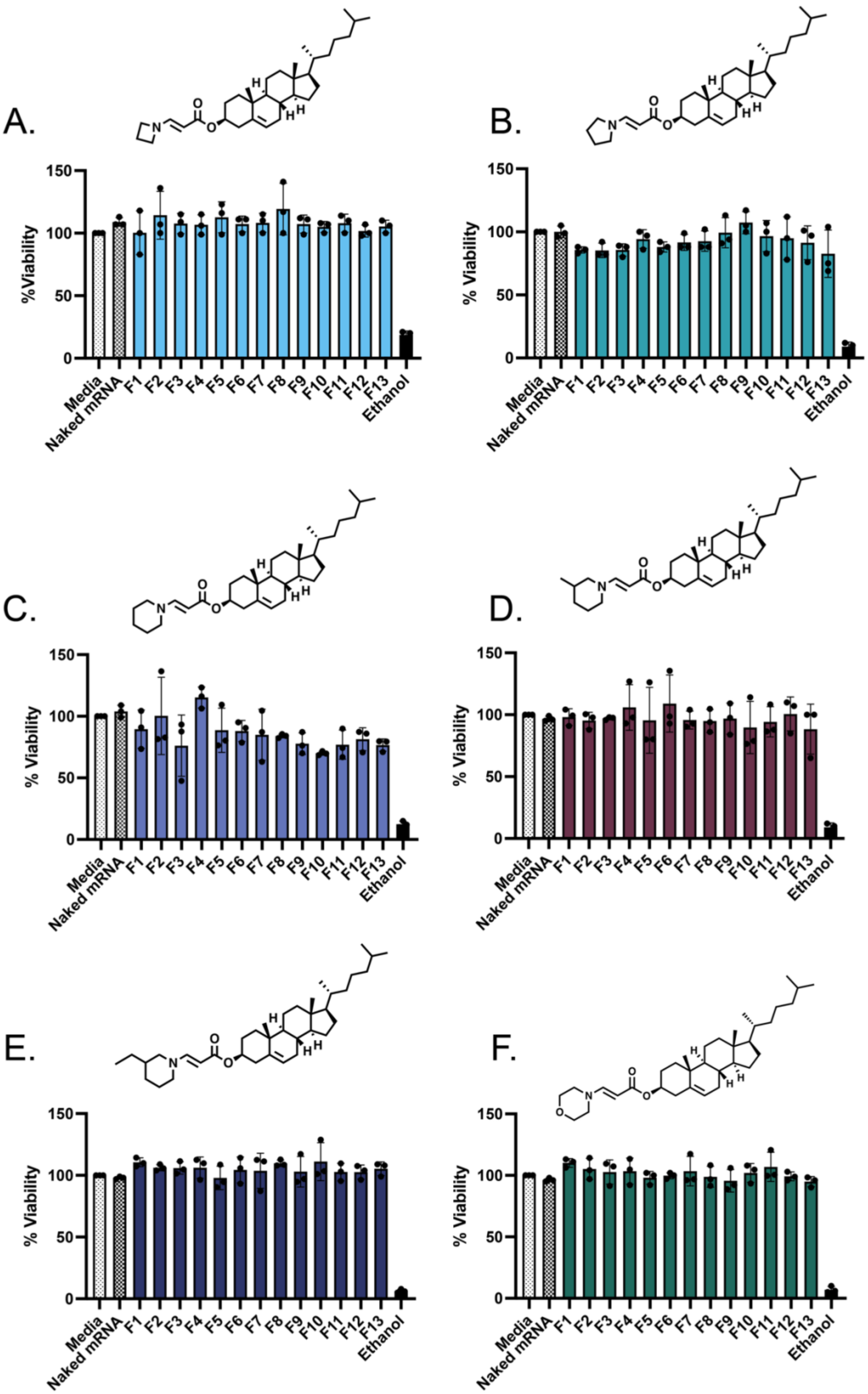

Supplementary Figure 12: (**a-f**) 24-hour viability for **CORE C1-6** on DC 2.4 cells at 50 ng FLuc mRNA p/ well. (All data represented as mean ± SD, n=3).

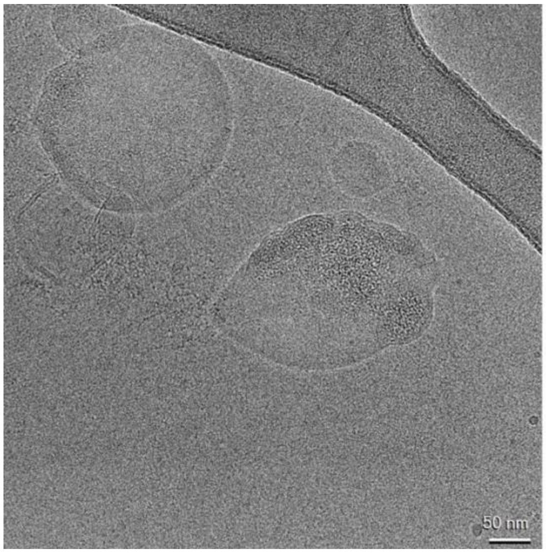

Supplementary Figure 13: Transmission electron microscopy (TEM) image of **CORE** LNP **C2:F13**.

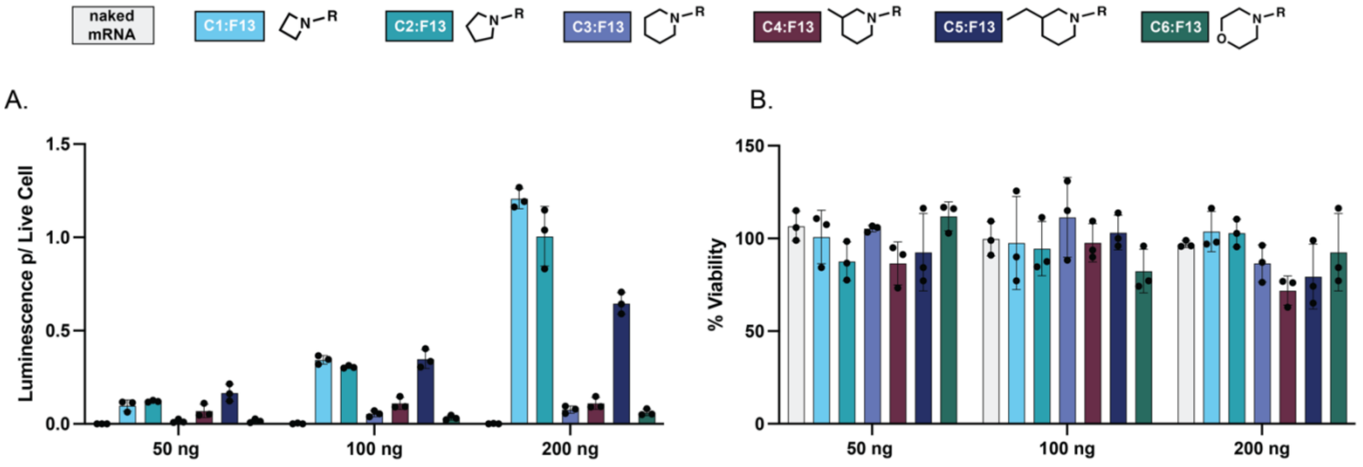

Supplementary Figure 14: (**a**) 24-hour FLuc expression of respective **CORE** LNPs treated on Jurkat cells at a dose of 50, 100, and 200 ng FLuc mRNA. (**b**) 24-hour viability of respective **CORE** LNPs treated on Jurkat cells at a dose of 50, 100, and 200 ng FLuc mRNA. (All data represented as mean ± SD, n=3).

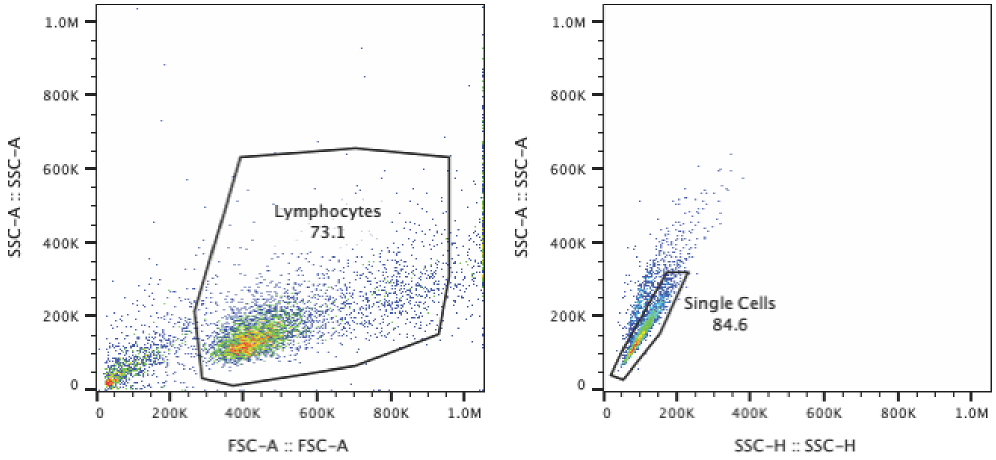

Supplementary Figure 15: Gating strategy utilized for cellular uptake and uptake pathway on DC 2.4 cells.

SM-102 LNP Formulation			
SM-102	Cholesterol	DSPC	DMG-PEG:2000
50%	38.5%	10%	1.5%
**Size (nm)**			
189 ± 2.5 nm (PDI=0.02)			
**Charge (mV)**			
0.15 ± 1			
**mRNA Encapsulation Efficiency (%)**			
89.5 ± 5			

Supplementary Figure 16: Formulation identity (denoted by molar ratio), size (nm), charge (mV), and mRNA encapsulation efficiency of SM-102 LNP.

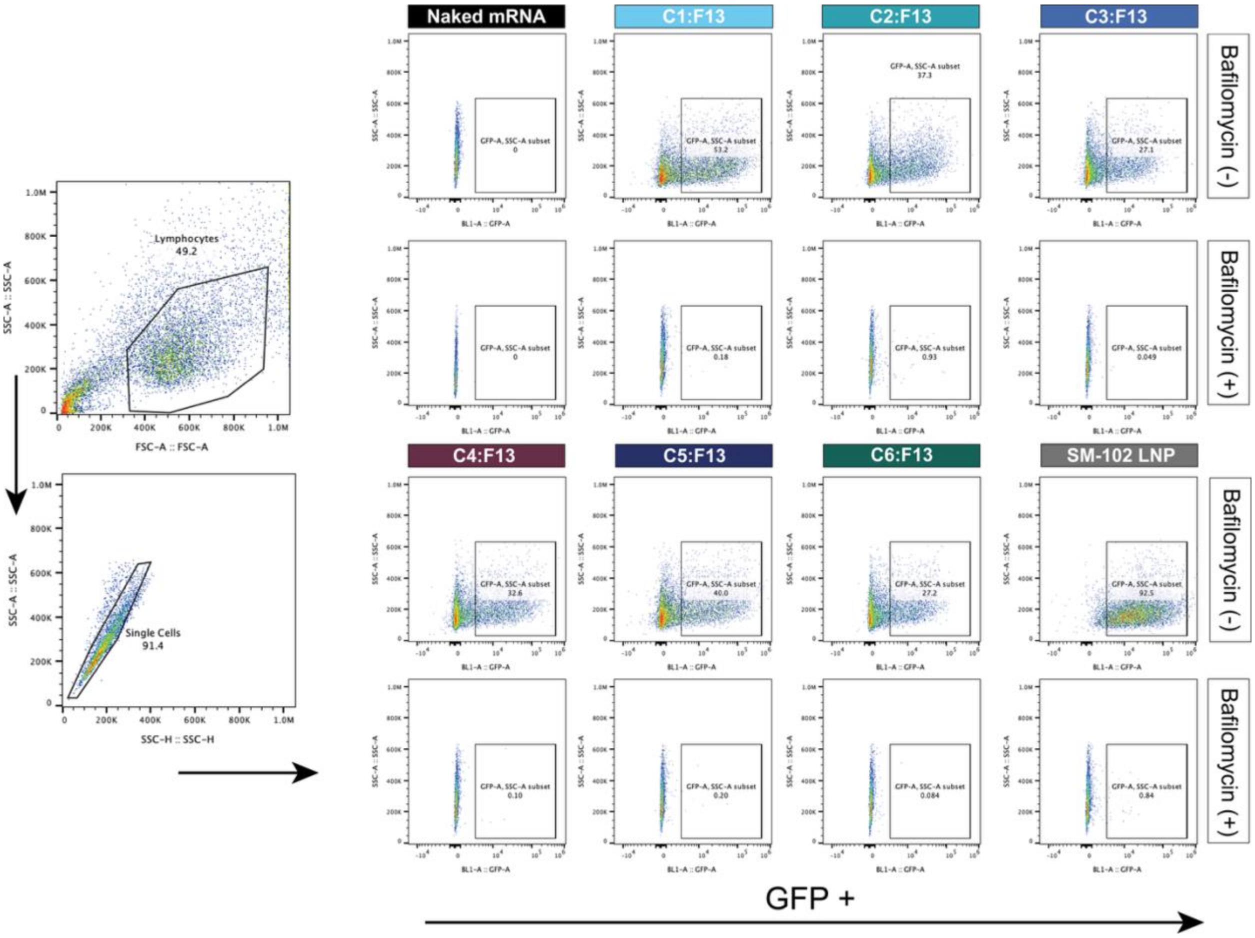

Supplementary Figure 17: Gating utilized to quantify gMFI of **CORE** LNP-mediated GFP expression with and without bafilomycin A_1 (inhibitor) treatment.

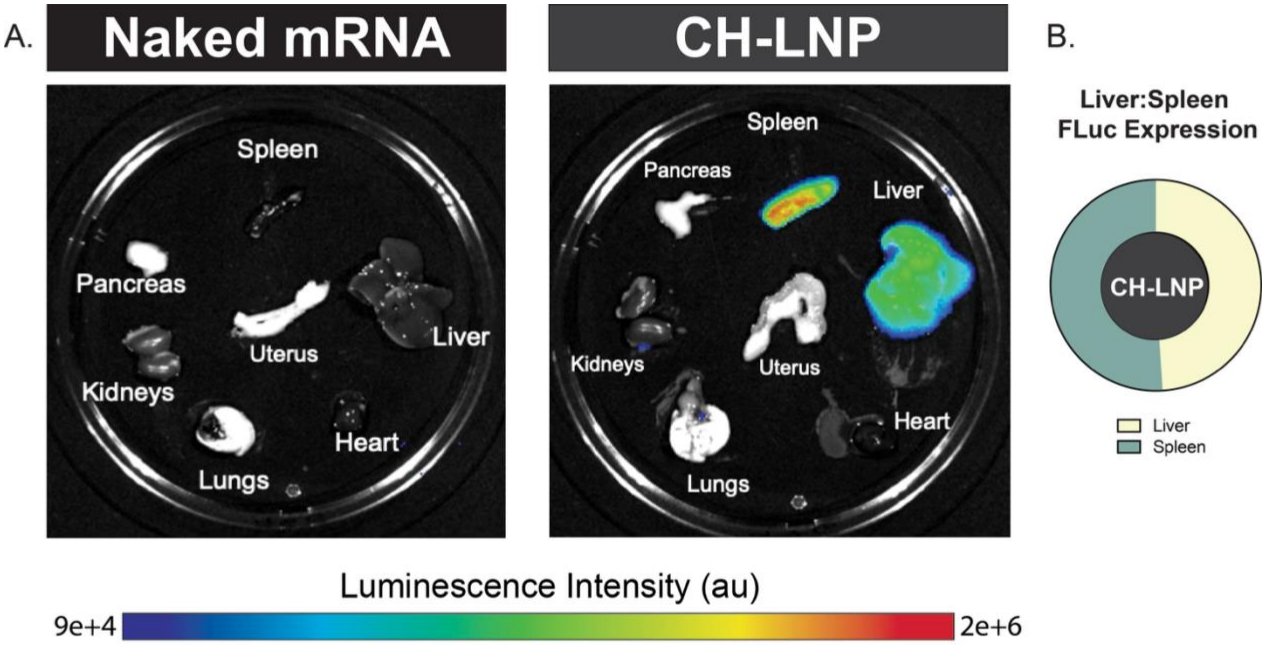

C.

CH-LNP<br>Formulation (F13)			
SM-102	Cholesterol	DOPE	14:0 PEG 1K
51%	28%	20.5%	0.5%
**Size (nm)**			
205 ± 5 nm (PDI:0.04)			
**Charge (mV)**			
0.03 ± 0.5			
**mRNA Encapsulation Efficiency (%)**			
75.4 ± 4			

Supplementary Figure 18: **(a)** Biodistribution imaging of CH-LNP after IV administration. **(b)** Liver-to-spleen ratio of FLuc expression of CH-LNP. **(c)** Formulation identity (denoted by molar ratio), size (nm), charge (mV), and mRNA encapsulation efficiency of CH-LNP.

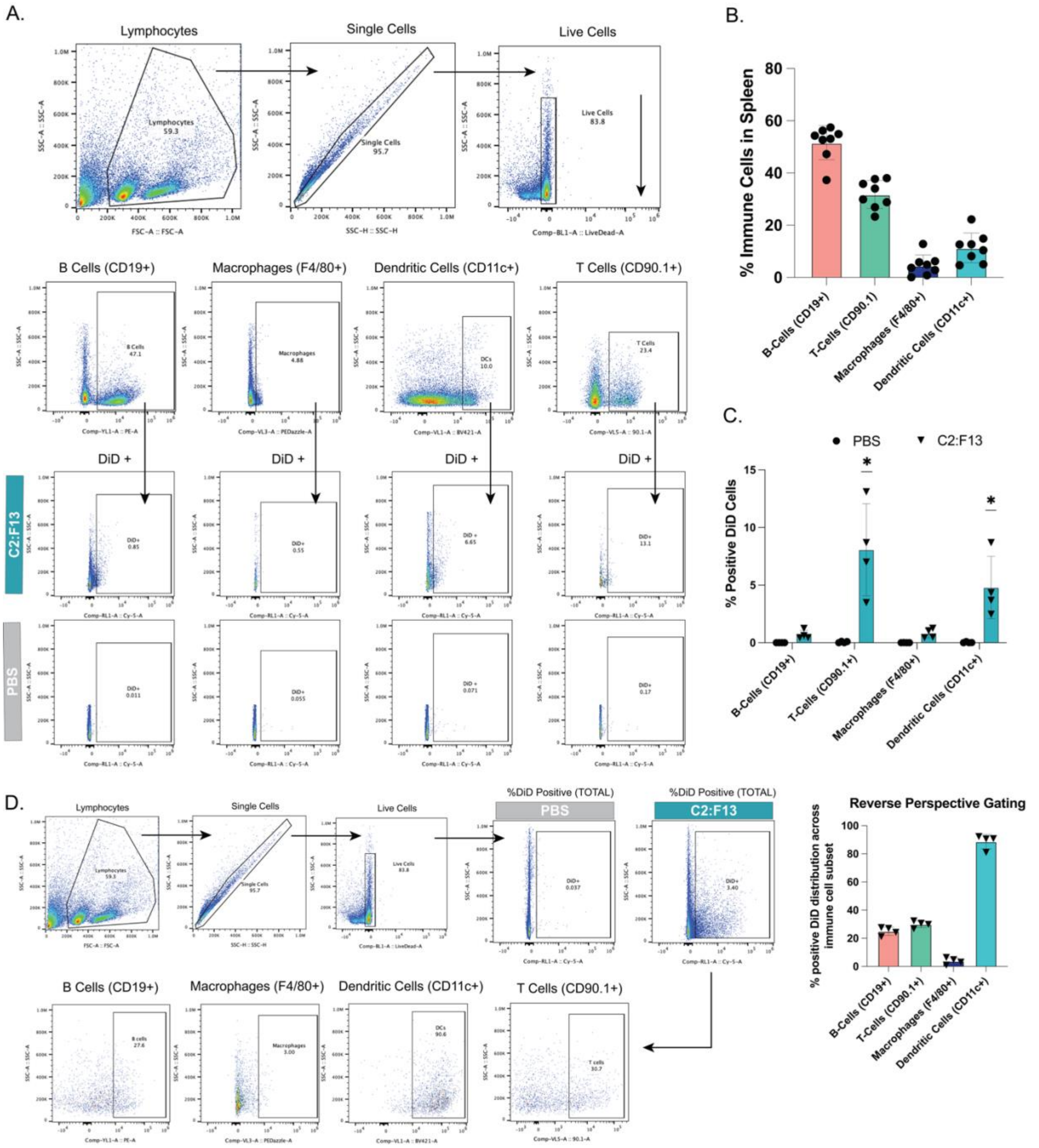

Supplementary Figure 19: (a) Gating strategy and representative flow cytometry data utilized to quantify **C2:F13** uptake in splenic immune cells. **(b)** Quantification of immune cell populations within C57BL/6 mice spleens (B cells, macrophages, dendritic cells, T cells). (All data represented as mean ± SD, n=8). **(c)** Percent quantification of DiD positive cells for **C2:F13** and PBS-treated mice. **(d**) Reverse complementary perspective gating analysis performed on **C2:F13** DiD uptake in which total immune cells positive for DiD were gated prior to assigning to immune cell subsets. (All data represented as mean ± SD, n=4 per group, *p<0.05 as compared to PBS treatment group using two-tailed t-test).

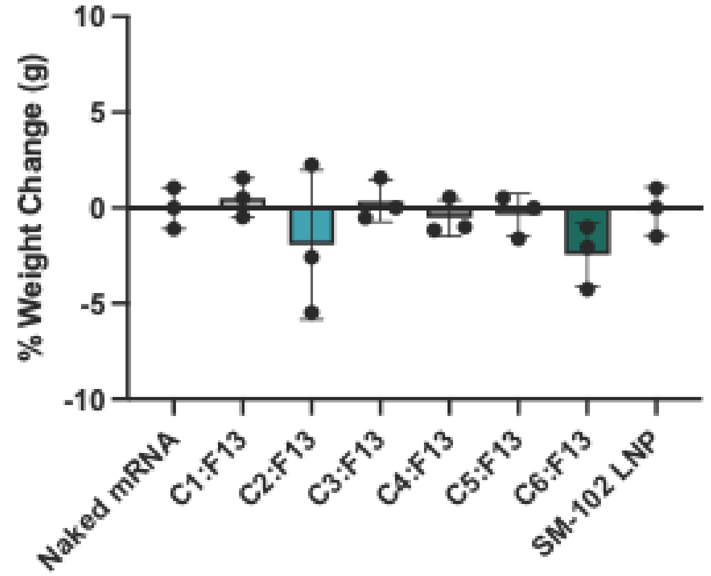

Supplementary Figure 20: % weight change of C57BL/6 mice 24 hours after treatment with **CORE** LNPs. (All data represented as the mean ± SD, n=3).

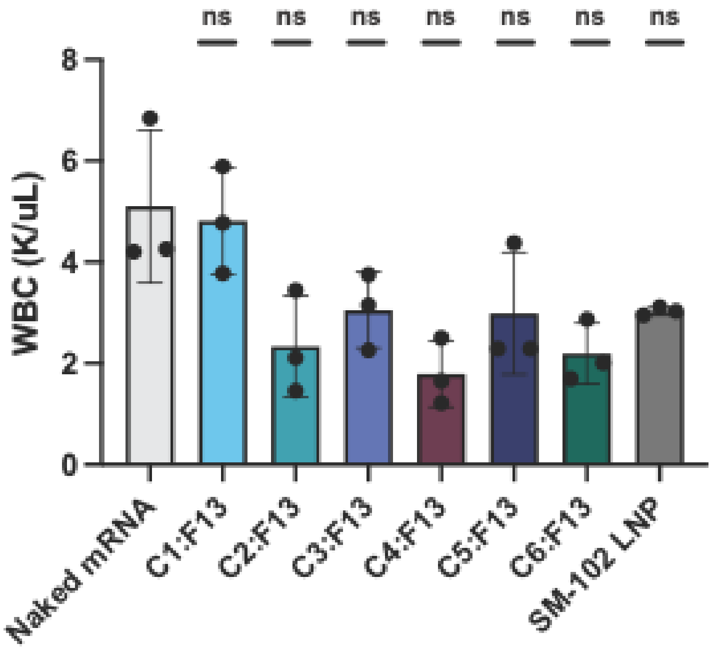

Supplementary Figure 21: White blood cell count measured 24 hours after treatment with **CORE** LNPs. (All data represented as the mean ± SD, n=3, ns = p>0.05 as compared to naked mRNA and SM-102 LNP treatment groups using one-way ANOVA, Dunnett's test).
